# m^6^Am-seq reveals the dynamic m^6^Am methylation in the human transcriptome

**DOI:** 10.1038/s41467-021-25105-5

**Published:** 2021-08-06

**Authors:** Hanxiao Sun, Kai Li, Xiaoting Zhang, Jun’e Liu, Meiling Zhang, Haowei Meng, Chengqi Yi

**Affiliations:** 1 State Key Laboratory of Protein and Plant Gene Research, School of Life Sciences, Peking University, Beijing, China; 2 Academy for Advanced Interdisciplinary Studies, Peking University, Beijing, China; 3 Peking-Tsinghua Center for Life Sciences, Peking University, Beijing, China; 4 Department of Chemical Biology and Synthetic and Functional Biomolecules Center, College of Chemistry and Molecular Engineering, Peking University, Beijing, China

**Keywords:** RNA, RNA sequencing, Methylation

## Abstract

N^6^,2′-*O*-dimethyladenosine (m^6^Am), a terminal modification adjacent to the mRNA cap, is a newly discovered reversible RNA modification. Yet, a specific and sensitive tool to directly map transcriptome-wide m^6^Am is lacking. Here, we report m^6^Am-seq, based on selective in vitro demethylation and RNA immunoprecipitation. m^6^Am-seq directly distinguishes m^6^Am and 5′-UTR N^6^-methyladenosine (m^6^A) and enables the identification of m^6^Am at single-base resolution and 5′-UTR m^6^A in the human transcriptome. Using m^6^Am-seq, we also find that m^6^Am and 5′-UTR m^6^A respond dynamically to stimuli, and identify key functional methylation sites that may facilitate cellular stress response. Collectively, m^6^Am-seq reveals the high-confidence m^6^Am and 5′-UTR m^6^A methylome and provides a robust tool for functional studies of the two epitranscriptomic marks.

## Abstract (web summary)

m^6^Am is a dynamic and reversible RNA modification found on the mRNA cap. Here the authors report m^6^Am-seq to directly distinguish m^6^Am from m^6^A and identify functional methylation sites.

## Introduction

More than 160 different chemical modifications have been found so far^1^. N^6^-methyladenosine (m^6^A) is the most abundant internal modification on mRNA and lncRNA in eukaryotes^2–4^. In addition to m^6^A, there exists another reversible modification in higher eukaryotes, called N^6^, 2′-*O*-dimethyladenosine (m^6^Am), which is precisely located at the first transcribed nucleotide and hence adjacent to the cap structure of mRNA^5,6^. A total of 50–80% of adenosine-starting mammalian mRNAs are believed to be m^6^Am modified^6^. It is catalyzed by PCIF1^7–10^, a protein that interacts with the phosphorylated CTD of RNAPII^11^, and could be removed by the m^6^A demethylase FTO^12,13^. Thus, m^6^Am is dynamically regulated by FTO and PCIF1, leading to the direction of the cap epitranscriptomics.

The development of transcriptome-wide sequencing technologies for various RNA modifications has greatly facilitated the field of epitranscriptomics^14^. The most widely used method for m^6^Am/m^6^A mapping relies on m^6^A antibodies, which do not discriminate between the two functionally distinct modifications^15,16^. While m^6^A is enriched around the stop codon, it is challenging to distinguish m^6^Am from 5′-UTR m^6^A, which constitutes a significant part of m^6^A methylome and has been shown to be functionally important in various biological processes^17–19^. There have been efforts aiming to distinguish the two modifications^8–10,20,21^; yet they suffer from the low efficiency of UV crosslinking, the inaccuracy of TSS annotation, and the limited activity of 5′ exonuclease, thus compromising the sensitivity and precision of m^6^Am detection. While the use of PCIF1 knockout (KO) cell lines has improved the confidence of m^6^Am detection^9,10^, it is an indirect approach and is incompatible for epitranscriptome analysis in human tissues and biological samples where genetic manipulation is challenging. Thus, a sensitive and direct method for transcriptome-wide m^6^Am identification is highly desired.

Here, we develop m^6^Am-seq to investigate the prevalence, topology, and dynamics of m^6^Am in the human transcriptome. m^6^Am-seq relies on an in vitro demethylation reaction that selectively removes m^6^Am while keeps m^6^A intact, thereby discriminating genuine m^6^Am at the mRNA cap and 5′-UTR m^6^A. Using m^6^Am-seq, we identify 2166 m^6^Am sites at base resolution from 1652 peaks and 1307 5′-UTR m^6^A peaks throughout the human transcriptome. Moreover, we show that m^6^Am and 5′-UTR m^6^A respond dynamically to heat shock and hypoxia conditions, and identify hundreds of stress-induced m^6^Am and 5′-UTR m^6^A peaks that could mediate adaptive response to the stressors. Altogether, we provide a tool to directly and selectively distinguish and profile m^6^Am and 5′-UTR m^6^A; we anticipate that m^6^Am-seq will pave the way for future functional studies of m^6^Am in various biological systems.

## Results

### A selective demethylation reaction to distinguish m^6^Am and m^6^A

m^6^Am-seq utilizes a selective demethylation reaction to achieve specific and sensitive detection of m^6^Am. While both m^6^Am and m^6^A are recognized by antibodies, we envisioned that the unique cap-adjacent structural context of m^6^Am could be selectively read out in vitro by demethylase (Supplementary Fig. 1a). To this end, we tested ALKBH5 and FTO, two known RNA demethylases^22,23^. We found that ALKBH5 does not act on m^6^Am, consistent with previous biochemical data^13^. However, it does not demethylate m^6^A to completion (~80%, Supplementary Fig. 1b), complicating subsequent analysis. In contrast, FTO prefers m^6^Am but demethylates both modifications with high efficiency (>98% and ~60% for m^6^Am and m^6^A, respectively), under recommended conditions from literature^13^ (Supplementary Fig. 1c).

**Fig. 1:**
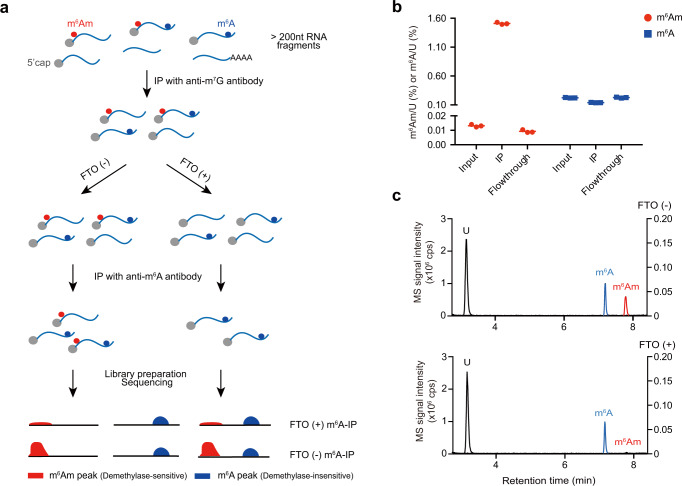
m^6^Am-seq combines RNA immunoprecipitation with an optimized demethylation reaction to enrich and distinguish m^6^Am and 5′-UTR m^6^A. **a** Scheme of m^6^Am-seq. Total RNA (>200 nt) was fragmented (input) and immunoprecipitated with cap-m^7^G antibody (m^7^G-IP). The cap structure-containing RNA was subjected to [FTO (+)] or [FTO (−)] treatment. The [FTO (+)] or [FTO (−)] samples were immunoprecipitated with an m^6^A antibody and sequenced for m^6^Am detection. The sequencing profiles of the [FTO (+) m^6^A-IP] and [FTO (−) m^6^A-IP] samples were normalized and compared. The “Demethylase-sensitive peaks” and “Demethylase-insensitive peaks” were identified. The demethylase-sensitive peaks bear the m^6^Am, while the demethylase-insensitive peaks bear the m^6^A (Online Methods). **b** The m^6^Am and m^6^A levels of input, m^7^G-IP, and flowthrough samples were quantified by LC–MS/MS, respectively. m^6^Am was significantly enriched (>100 fold) by cap-m^7^G antibody while m^6^A level decreased mildly. Values represent mean ± SD (*n* = 3 independent samples in “Input”, “IP” and “Flowthrough” respectively). **c** The m^6^Am and m^6^A levels of [FTO (+)] and [FTO (−)] samples were quantified by LC–MS/MS, respectively. >95% m^6^Am was demethylated and m^6^A was negligibly altered under optimized demethylation conditions. The chromatograms of U (black) and m^6^Am (red) are scaled to the left *Y* axis, and the chromatograms of m^6^A (blue) are scaled to the right *Y* axis. Source data are provided as a Source Data file.

Neither of the demethylation reactions would be good enough to distinguish m^6^Am and m^6^A in their present forms. The ideal condition would remove one modification to completion while leaving another intact. FTO and ALKBH5 are Fe(II)/2-KG-dependent dioxygenases, whose in vitro demethylation activity is promoted by l-ascorbic acid^22,23^. As a reducing agent, it is capable of reducing redox-active metals such as iron, thereby increasing the pro-oxidant chemistry of these metals^24^. We thus hypothesized that by omitting l-ascorbic acid from our conditions, we might be able to modulate the in vitro activity of FTO so that it only acts on the preferred, cap-adjacent m^6^Am but not the internal m^6^A. To our delight, after removing l-ascorbic acid and further optimizing the demethylation conditions, >95% of m^6^Am was removed without significantly affecting the m^6^A level (Fig. 1c). We further verified the selectivity of our demethylation condition using a dually modified probe containing both cap m^6^Am and internal m^6^A, which were 76 nt apart from each other (Supplementary Fig. 1d). Thus, we identified a demethylation reaction that is specific for m^6^Am.

### m^6^Am-seq directly detects m^6^Am

To map the m^6^Am in the human transcriptome, we developed m^6^Am-seq, which combines the selective demethylation reaction with RNA immunoprecipitation (Fig. 1). Two key RNA immunoprecipitation procedures are utilized, which are a cap-m^7^G IP and an m^6^A IP step. Because the global m^6^A level is around 20-fold higher than that of m^6^Am in total RNA (Supplementary Fig. 1e), and the vast majority of m^6^Am is localized in mRNA cap (Supplementary Fig. 1f), we used the cap-m^7^G IP step to enrich for m^6^Am and deplete m^6^A that is mostly found in the 3′-UTR. As expected, immunoprecipitation with a cap-m^7^G antibody for fragmented RNA resulted in more than 100-fold enrichment of m^6^Am and a simultaneous 2-fold reduction of m^6^A (Fig. 1b). Moreover, ~90% of peaks are located at and near 5′-UTR, suggesting 5′-end of mRNAs were successfully and specifically enriched using size selected total RNA (>200 nt) as input, without the prerequisite to purify mRNA (Supplementary Fig. 2a).

The immunoprecipitated RNA was then subjected to the selective demethylation reaction or mock treatment, which was followed by the second IP step utilizing an m^6^A antibody (Fig. 1a). The FTO (−) sample would contain both m^6^Am and 5′-UTR m^6^A signals, while the FTO (+) sample would contain only m^6^A. By comparing the methylome profiles of the two samples, we were able to detect m^6^Am, which is sensitive to demethylase treatment, from 5′-UTR m^6^A, which is insensitive and conserved in the two samples. Indeed, we observed a bimodal-like distribution of fold change of peak intensity: peaks on the right side experienced reduced intensity upon demethylase treatment, suggesting they are demethylase-sensitive m^6^Am modification; while peaks on the left were not significantly altered or even slightly increased, suggesting they are retained 5′-UTR m^6^A peaks (Supplementary Fig. 2b, c). Thus, m^6^Am-seq selectively distinguishes the two modifications.

### m^6^Am-seq detects transcriptome-wide m^6^Am and 5′-UTR m^6^A

**Fig. 2:**
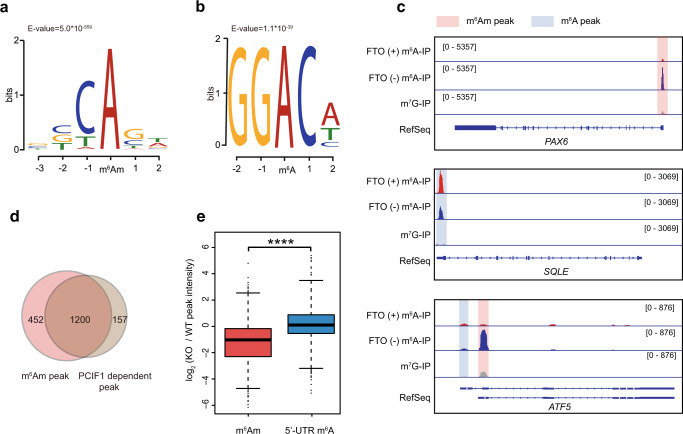
m^6^Am-seq uncovers the high-confidence m^6^Am and 5′-UTR m^6^A methylation in the human transcriptome. Motif analysis of m^6^Am peaks (**a**) and 5′-UTR m^6^A peaks (**b**) identified by m^6^Am-seq. **c** Representative views of typical m^6^Am peaks and 5′-UTR m^6^A peaks on mRNA. The methylation peak in the 5′-UTR of *PAX6* was lost in “FTO (+) m^6^A-IP” samples, suggesting a demethylase-sensitive m^6^Am modification (top); the methylation peak in the 5′-UTR of *SQLE* retained well in “FTO (+) m^6^A-IP” samples, suggesting a demethylase-insensitive m^6^A modification (middle); while multiple isoforms of *ATF5* were differentially marked by m^6^Am and 5′-UTR m^6^A (bottom). Pink and blue background colors denote m^6^Am and m^6^A signals, respectively. **d** Venn diagram showing the overlap between m^6^Am peaks identified directly by m^6^Am-seq and m^6^Am peaks identified indirectly by m^6^A IP using PCIF1-KO samples. **e** Boxplot showing that the peak intensity of m^6^Am (red, *n* = 1652), but not that of m^6^A (blue, *n* = 1307), was significantly reduced upon PCIF1 depletion. Statistical significance of the difference was determined by unpaired two-sided Mann–Whitney *U*-test. ****P < 2.2e^−16^. Boxes represent 25th–75th percentile (line at median) with whiskers at 1.5*IQR. Source data are provided as a Source Data file.

We next applied m^6^Am-seq to characterize m^6^Am and 5′-UTR m^6^A methylome in HEK293T cells. We found that m^6^Am-seq results are highly correlated between biological replicates (correlation coefficient = 0.9821, Supplementary Fig. 2d), demonstrating its robustness. We identified 1652 and 1307 high-confidence m^6^Am and 5′-UTR m^6^A peaks from 1635 and 1297 genes, exhibiting the characteristic BCA (B = C/U/G) and GGACH (H = A/C/U) motif, respectively (Fig. 2a, b and Supplementary Data 1, 2). m^6^Am shows the highest enrichment at annotated TSSs, while the summit of m^6^A is clearly within 5′-UTR; the peak density of m^6^Am is also 3-fold higher than that of 5′-UTR m^6^A, demonstrating that m^6^Am is more concentrated than m^6^A around the TSSs (Supplementary Fig. 3a, b). Representative methylation profiles are shown in Fig. 2c. In one example, the *ATF5* gene has multiple isoforms with distinct TSS and alternative first exons; we observed m^6^Am modification for one transcript and 5′-UTR m^6^A for another transcript (Fig. 2c), suggesting differential cis-regulatory elements in regulating the epitranscriptomic status of different transcripts from the same gene. Gene ontology (GO) terms analysis showed that transcripts containing m^6^Am and 5′-UTR m^6^A are most strongly enriched in cell cycle and RNA metabolism, respectively, suggesting their differential functions (Supplementary Fig. 3c, d).

To further demonstrate the accuracy of m^6^Am-seq, we applied standard m^6^A IP experiments to PCIF1-KO HEK293T cells. Based on the reduction of signals compared to WT cells, we detected 1357 PCIF1-dependent peaks; notably, 1200 out of the 1357 peaks were identified by m^6^Am-seq (Fig. 2d and Supplementary Fig. 3e), suggesting the high confidence of m^6^Am-seq. The remaining peaks all exhibited m^6^Am-like property (i.e., sensitive to demethylation) in m^6^Am-seq but were not called due to the stringent cutoff we used. On the other hand, 5′-UTR m^6^A peaks identified by m^6^Am-seq were not affected upon PCIF1 depletion (Fig. 2e and Supplementary Fig. 3f), further demonstrating the specificity of m^6^Am-seq. Taken together, these results prove that m^6^Am-seq identifies m^6^Am with high confidence. It is also worth mentioning that m^6^Am-seq is applicable for m^6^Am detection in tissues or cell populations where PCIF1 KD or KO is not feasible.

### Comparisons of existing m^6^Am detection methods

**Fig. 3:**
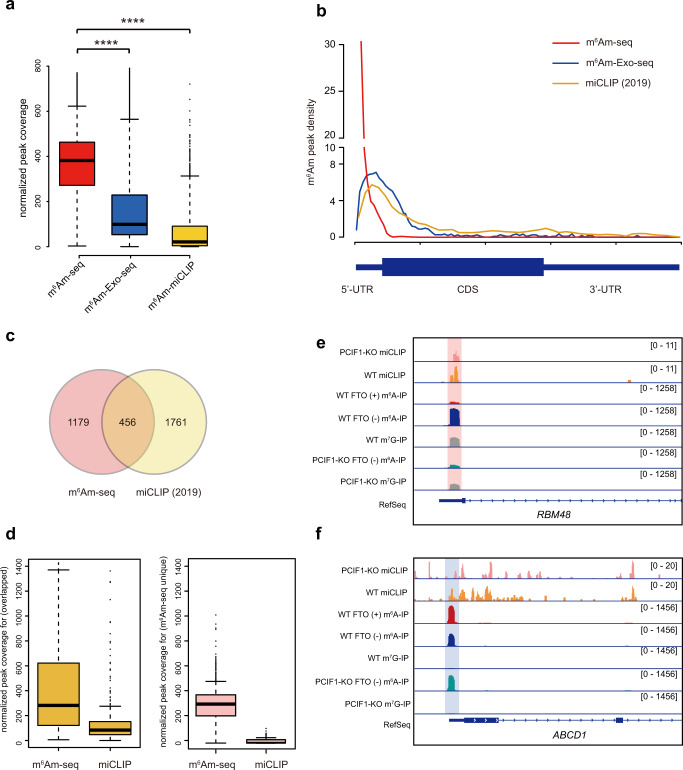
Comparison of m^6^Am methylome detected by m^6^Am-seq and existing m^6^Am detection methods. **a** Boxplot showing that the normalized peak coverage by m^6^Am-seq (red, *n* = 1652), m^6^Am-Exo-seq (blue, *n* = 3169), and miCLIP (yellow, *n* = 2217). Statistical significance of the difference was determined by unpaired two-sided Mann–Whitney *U*-test. ****P < 2.2e^−16^ for m^6^Am-seq vs m^6^Am-Exo-seq, m^6^Am-seq vs miCLIP. Boxes represent 25th–75th percentile (line at the median) with whiskers at 1.5*IQR. **b** Metagene profiles of m^6^Am distribution identified by the three sequencing methods. Each segment was normalized according to its average length in RefSeq annotation. **c** Venn diagram showing the overlap of m^6^Am-marked genes by m^6^Am-seq and by miCLIP. **d** Boxplot showing the normalized peak coverage of shared m^6^Am peaks (yellow, *n* = 456, identified in both m^6^Am-seq and miCLIP) and m^6^Am-seq-unique m^6^Am peaks (pink, *n* = 1179) in the m^6^Am-seq and miCLIP data sets, respectively. Boxes represent 25th–75th percentile (line at the median) with whiskers at 1.5*IQR. **e** An example of m^6^Am (in the *RBM48* gene), which was missed by miCLIP due to its limited sensitivity but is clearly identified by m^6^Am-seq. The m^6^Am peak is lost in PCIF1-KO data sets, further proving the detection confidence of m^6^Am-seq. Pink background color denotes m^6^Am signal. **f** An example of mis-annotated m^6^Am (in the *ABCD1* gene) by miCLIP. This peak is not affected by FTO treatment nor PCIF1-KO, showing that it is a 5′-UTR m^6^A modification. Blue background color denotes m^6^A signal. Source data are provided as a Source Data file.

We then compared m^6^Am-seq results with m^6^Am-Exo-seq and miCLIP^9,10^. Under normalized sequencing depth, the averaged peak coverage of m^6^Am-seq (~387) was ~4× and 21× higher than that of m^6^Am-Exo-seq (~95) and miCLIP (~18) (Fig. 3a). In addition, m^6^Am peaks by m^6^Am-seq exhibit a strong tendency towards the annotated TSS, in comparison to a modest enrichment within 5′-UTR by miCLIP and m^6^Am-Exo-Seq (Fig. 3b). Thus, m^6^Am-seq outperforms existing methods in m^6^Am detection.

Because m^6^Am-seq and miCLIP can both detect m^6^Am and m^6^A, we performed comparisons of the two methods^10,20^ (Fig. 3c and Supplementary Fig. 4a, b). We used the recent miCLIP data relying on PCIF1-KO for detailed comparison^10^. Approximately 21% (456/2217) of m^6^Am marked genes by miCLIP overlap with that of m^6^Am-seq (Fig. 3c). We found that miCLIP data sets showed very low coverage, providing an explanation for the limited sensitivity of miCLIP (Fig. 3d, e). While it is possible that the stringent cutoff of m^6^Am-seq may favor specificity over sensitivity, a significant portion of miCLIP-identified m^6^Am peaks could be mis-annotated and are instead 5′-UTR m^6^A peaks (Fig. 3f).

It was proposed that m^6^Am controls mRNA stability^12^, based on the m^6^Am methylome identified by miCLIP. Given the observation that miCLIP may mis-annotate the two modifications, we compared the expression of m^6^Am-containing genes, which are identified by m^6^Am-seq, in wild-type and PCIF1-KO cells. Different from the previous conclusion^12^, we found that the loss of m^6^Am did not alter the expression of their host mRNA (Supplementary Fig. 4c).

### m^6^Am-seq detects m^6^Am at single-base resolution

**Fig. 4:**
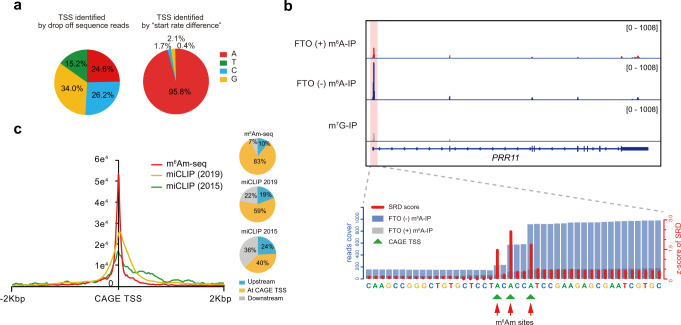
m^6^Am-seq detects m^6^Am methylome at single-base resolution. **a** Nucleotide proportion of TSS identified by the sudden drop-off of sequencing reads (left) and by “start rate difference” (SRD) metrics. **b** A representative view of three single m^6^Am sites on the transcripts of *PRR11*, which were all supported by CAGE data. Three adenosine residues with a high SRD score (red bar) were defined as m^6^Am sites, which overlapped exactly with CAGE sites (green triangles). Each segment was normalized according to its average length in RefSeq annotation. Pink background color denotes m^6^Am signal. **c** A metaplot showing that the vast majority of m^6^Am sites by m^6^Am-seq overlapped with CAGE sites, suggesting that these m^6^Am sites are indeed TSSs. Compared with miCLIP, m^6^Am sites identified by m^6^Am-seq were much closer to the CAGE TSSs. Two miCLIP data sets were used for comparison: an m^6^Am list reported in 2015 ^20^ and an updated m^6^Am list in 2019 ^10^, based on PCIF1-KO. Source data are provided as a Source Data file.

Because m^6^Am is the first transcribed nucleotide, one would expect to identify m^6^Am at base resolution by searching for A-starting transcripts (i.e., those showing sudden drop-off of sequencing reads at the 5′ end). However, even when we used a library preparation procedure that preserves the first transcribed nucleotide, we did not observe an enrichment of A nucleotide in the m^6^Am/m^6^A enriched FTO (−) data set (Fig. 4a), suggesting that its TSS location alone is not sufficient to detect m^6^Am sites. Thus, we defined a “start rate difference” score (SRD score) for each nucleotide to quantify sequencing reads beginning from it (see “Methods” section). Application of these metrics immediately resulted in accurate identification of the m^6^Am site in synthetic spike-in RNAs (Supplementary Fig. 5a) and ~95.8% of A nucleotides in the m^6^Am/m^6^A enriched FTO (−) data set (Fig. 4a). In total, we identified 2166 m^6^Am sites from 1459 genes (Supplementary Data 3). For instance, mRNA-coding gene *PRR11* and a lncRNA *LOC101929147* both have alternative TSSs that start with an A nucleotide, and we showed that all these transcripts are marked by m^6^Am (Fig. 4b and Supplementary Fig. 5b). In addition, while m^6^Am is associated with a BCA motif, our single-base m^6^Am methylome revealed ~34% non-BCA m^6^Am sites (Supplementary Fig. 6a). We found also a weaker signal of GRO-seq and H3K27ac ChIP-seq for the non-BCA m^6^Am sites, but no difference in H3K4me3, H3K4me1, and H3K9me3 signals (Supplementary Fig. 6b–f). Altogether, these data indicate that m^6^Am-seq enables m^6^Am detection at single-base resolution.

To further characterize the accuracy of the identified m^6^Am sites, we performed the cap-analysis of gene expression (CAGE) experiments and compared the m^6^Am sites with annotated and CAGE-identified TSSs. We found that most of the m^6^Am sites are present downstream of the RefSeq-annotated TSSs, with only 14% of m^6^Am sites mapped at the annotated TSSs (Supplementary Fig. 7a). This observation suggested that existing methods based on annotated TSSs information can be inaccurate for m^6^Am mapping. For instance, the m^6^Am site of *TMET241* would have been located internally within 5′-UTR if the annotated TSSs were used (Supplementary Fig. 7b); and two m^6^Am peaks are clearly identified for *RAB5A*, which has only one annotated TSS (Supplementary Fig. 7c). We next plotted the distance from m^6^Am sites to their nearest CAGE TSS sites, and observed that the vast majority of m^6^Am sites overlapped with CAGE TSSs (Fig. 4c), suggesting that these m^6^Am sites are indeed TSSs. Compared with miCLIP^10,20^, m^6^Am sites by m^6^Am-seq were much closer to the CAGE TSSs (Fig. 4c). These data highlight the reliability of m^6^Am-seq in obtaining high-confidence m^6^Am sites.

### Heat shock induces dynamic m^6^Am and 5′-UTR m^6^A methylations

**Fig. 5:**
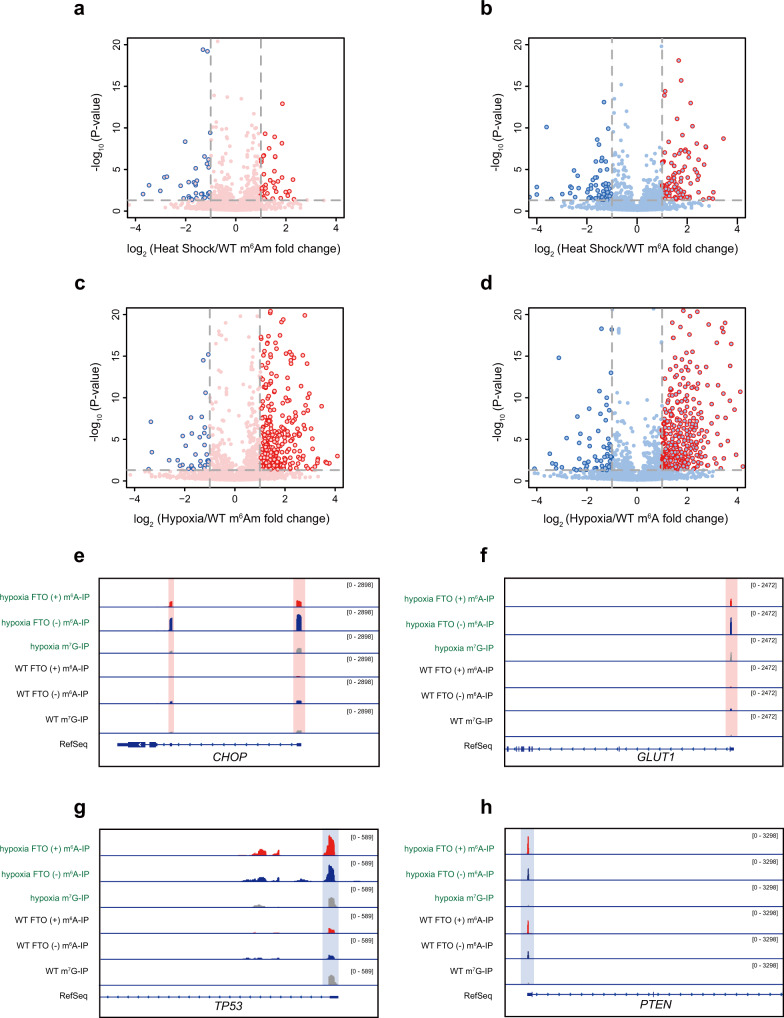
m^6^Am and 5′-UTR m^6^A are dynamically regulated by different stress conditions. **a**–**d** Volcano plot showing stress-induced m^6^Am and 5′-UTR m^6^A: heat shock-inducible m^6^Am peaks (**a**) and 5′-UTR m^6^A peaks (**b**), and hypoxia-inducible m^6^Am peaks (**c**), and 5′-UTR m^6^A peaks (**d**). Red circles denote stress-upregulated peaks; blue circles denote stress-downregulated peaks. Representative views of elevated m^6^Am peaks in *CHOP* (**e**) and *GLUT1* (**f**) under hypoxia stress. Representative views of elevated 5′-UTR m^6^A peaks in *TP53* (**g**) and *PTEN* (**h**) under hypoxia stress. Pink and blue background colors denote m^6^Am and m^6^A signals, respectively.

Stress-induced RNA modification is an important feature of dynamic epitranscriptomic regulation under physiological conditions^15,25–27^. For instance, it has been shown that in response to heat shock stress, m^6^A deposited to the 5′-UTR of newly transcribed mRNAs promotes cap-independent translation initiation, providing a mechanism of selective mRNA translation during general translation inhibition^17,19^. In light of this, we applied m^6^Am-seq to characterize the dynamic m^6^Am and 5′-UTR m^6^A methylome under heat shock conditions. We identified 45 upregulated m^6^Am peaks and 90 5′-UTR m^6^A peaks, as well as 36 and 67 downregulated m^6^Am and 5′-UTR m^6^A peaks (Fig. 5a, b and Supplementary Fig. 8a, b and Supplementary Fig. 9a–d and Supplementary Data 4). Among them, we found key heat shock-associated transcripts with elevated 5′-UTR m^6^A: notable examples include *HSPA1A*, *HSPA1B,* and *DNAJB1* (Supplementary Fig. 10a, b), whose transcripts undergo m^6^A-dependent but cap-independent selective translation^17^. These results by m^6^Am-seq accurately recapitulated heat shock-induced, functional 5′-UTR m^6^A methylation.

Unexpectedly, a subset of transcripts previously proposed to rely on m^6^A-dependent translation under heat shock^17^ did not contain m^6^A modification in the 5′-UTR; instead, m^6^Am-seq reveals that they are dynamically modified by m^6^Am (Supplementary Fig. 10c). For instance, using a method that does not distinguish between m^6^Am and 5′-UTR m^6^A, upregulation of 5′-UTR m^6^A level in *EGR1* was believed to mediate the stress-induced selective translation. However, using m^6^Am-seq, we found that *EGR1* contained m^6^Am rather than 5′-UTR m^6^A (Supplementary Fig. 10d). Therefore, cap-independent translation of such transcripts including *EGR1* is unlikely to be mediated by m^6^A. Whether or not m^6^Am, which is located at TSS and immediately after the m^7^G cap, can also promote stress-induced selective translation would be of interest for future studies. Overall, m^6^Am-seq provides accurate methylome maps to enable the study of the physiological roles of the two RNA modifications.

### Dynamic m^6^Am and 5′-UTR m^6^A methylome under hypoxia conditions

We next determined whether m^6^Am and 5′-UTR m^6^A methylation can be dynamically regulated by hypoxia. We were able to identify 362 and 638 elevated m^6^Am and 5′-UTR m^6^A peaks respectively, with 31 and 63 decreased m^6^Am and 5′-UTR m^6^A peaks (Fig. 5c, d and Supplementary Fig. 8c, d and Supplementary Data 5). While some of the hypoxia-specific genes show inducible m^6^Am and 5′-UTR m^6^A modifications, many transcripts with unaltered expression levels are also dynamically modified (Supplementary Fig. 9e–h). Overall, we observed a more evident increase of m^6^Am and m^6^A methylome under hypoxia conditions than heat shock conditions.

To cope with hypoxia, mammals have evolved key adaptive mechanisms including cellular adaptations of protein synthesis, energy metabolism, mitochondrial respiration, lipid, and carbon metabolism as well as nutrient acquisition^28^. Given the dramatic change of m^6^Am level, we examined whether dynamic m^6^Am may participate in the adaptive mechanisms during the hypoxia condition. Intriguingly, our GO analysis showed that genes with elevated m^6^Am levels are linked to the endoplasmic reticulum (ER) stress-response regulation (Supplementary Fig. 11a). For instance, it has been documented that transcriptional and translational upregulation of both *CHOP* and *ATF3* are important to activate the expression of downstream genes in response to stress^29,30^; in line with this, we observed significantly elevated m^6^Am levels in their mRNAs (Fig. 5e and Supplementary Data 5). In addition to ER stress, oxygen deprivation also reprograms intracellular metabolism^31^. For instance, hypoxia can enhance the mRNA and protein levels of HIF1 target gene *GLUT1* to achieve glucose transport^31^; in agreement with this, we also observed increased m^6^Am level in the *GLUT1* mRNA (Fig. 5f). Thus, we identified a dynamic m^6^Am program in key transcripts that are utilized by cells to adapt to hypoxia.

Distinct from the m^6^Am-marked genes, GO analysis of genes with elevated 5′-UTR m^6^A indicated an enrichment of the p53 signaling pathway (Supplementary Fig. 11b). In fact, hypoxia has frequently been described to be a p53 inducer^32^. Along this line, p53 and many of its downstream genes, such as *MDM2*, *PTEN*, *PIDD1*, etc., were found to contain elevated 5′-UTR m^6^A under hypoxia stress (Fig. 5g, h and Supplementary Data 5). Since induced 5′-UTR m^6^A by heat shock has been shown to enable cap-independent translation, it is possible that protein synthesis of the p53 signaling pathway could be modulated by the stress-induced m^6^A. Altogether, our results suggest that dynamic m^6^Am and m^6^A as key components of the adaptive response to hypoxia.

## Discussion

More than 40 years have passed since the initial documentation of m^6^Am was made^5,6^. The recent discovery of its “eraser” and “writer” has led to a renewed interest in this reversible modification^33^. Yet its biological functions remain enigmatic, due to a lack of sensitive methods that detect the modification at the transcriptome-wide level. Here, we report m^6^Am-seq, which enables specific and robust profiling of m^6^Am in the human transcriptome.

The development of m^6^Am-seq is inspired by existing epigenomic and epitranscriptomic methods utilizing biologically important demethylases. For instance, TAB-Seq^34^ and TAPS^35^ rely on the TET proteins, which oxidize 5-methylcytosine (5mC), to detect 5mC and its oxidative derivatives in the genome. In DM-tRNA-seq^36^, ARM-seq^37^, and m^1^A-MAP^38^, the RNA/DNA demethylase AlkB has been used to map N^1^-methyladenosine (m^1^A) in tRNA and mRNA. In this study, we expand the available toolbox by repurposing FTO to detect m^6^Am and m^6^A, two dynamic and reversible epitranscriptomic marks. Different from the existing methods that use demethylases to indiscriminately remove all substrates, we manipulated the in vitro activity of FTO to selectively demethylate just one of the two cognate substrates. This is achieved by the discovery of a controlled in vitro demethylation reaction, which omits the key cofactor l-ascorbic acid from the biochemical condition. Owing to its capacity to reduce Fe^3+^ to the catalytically active Fe^2+^, l-ascorbic acid can enhance the in vitro demethylation activity^24,39^. By leaving out l-ascorbic acid from the in vitro reactions, FTO still possesses a high m^6^Am demethylation activity while minimally affects the level of m^6^A, hence allowing selective detection of m^6^Am.

Interestingly, the in vivo bioavailability of ascorbate, the dominant form of vitamin C under physiological pH conditions, has been demonstrated to impact the epigenome via TETs and JmjC domain-containing demethylases, ultimately leading to phenotypic changes in development, aging, cancer and other diseases^24^. It remains to be seen whether or not the deficiency of vitamin C might alter m^6^Am or m^6^A methylome and hence epitranscriptome reprogramming inside of cells.

Except for the selective demethylation condition omitting l-ascorbic acid, major steps of m^6^Am-seq are well-known meRIP protocols. Hence, m^6^Am-seq is straightforward and highly reproducible. Its high sensitivity and accuracy are also enabled by the combination of cap-m^7^G IP and m^6^A IP, which builds on the knowledge that m^6^Am is adjacent to the m^7^G cap. It should be noted that m^6^Am-seq does not detect internal m^6^Am, although the amount of the internal m^6^Am in mRNAs seems negligible. Only one internal m^6^Am site in U2 snRNA has been documented so far. In m^6^Am-seq, we used a demethylation reaction to distinguish m^6^Am from m^6^A, other selective chemistry could be exploited for the determination of additional modifications. For instance, dNTP-sensitivity during reverse transcription^40^ and peroxidate oxidation^41^ could be combined with cap-m^7^G IP to detect ribose methylation at the first and second transcribed nucleotides of mRNA. In addition, given the diversity of RNA cap structures^42,43^, including NAD+, NADH, desphospho-CoA, and 2,2,7-trimethylguanosine, tailored combination of IP enrichment and a discrimination procedure, as demonstrated in m^6^Am-seq, could have broad applications in analyzing the epitranscriptomic state of the transcriptome.

m^6^Am-seq distinguishes m^6^Am from m^6^A and provides a high-confidence m^6^Am methylome. While a previous study used miCLIP for m^6^Am identification and has indicated a role of m^6^Am in controlling mRNA stability^12^, this conclusion was disputed by more recent studies led by independent groups^7,9,13^. Using m^6^Am-seq, we corroborated the latter studies and found that m^6^Am does not affect mRNA stability. Because miCLIP does not separate well m^6^Am from m^6^A, it is likely that the observed effect in mRNA stability is caused by m^6^A^13^, which has been shown to increase mRNA stability when recognized by certain m^6^A readers^44^. Nevertheless, such disagreement underscores the importance of a specific method to accurately map m^6^Am for future functional studies.

We showed that m^6^Am is dynamically regulated by heat shock and hypoxia, hinting that m^6^Am is important for cellular stress response. While a specific role of m^6^Am remains to be elucidated, we found that many well-known stress-induced genes exhibiting elevated protein levels showed increased m^6^Am methylation levels upon stimuli. Such a positive correlation appears to be the opposite in normal human tissues^21^; nevertheless, this does not rule out the possibility of a unique role of m^6^Am under stress. Consistent with the positive association, ribosome profiling experiments also revealed a positive role of m^6^Am in translation^7^. Given that 5′-UTR m^6^A has been demonstrated to mediate cap-independent translation^17,19^, it is tempting to speculate that m^6^Am plays a role in stress-induced mRNA translation.

Whether or not m^6^Am may participate in cap-independent translation is an open question. It is known that the mRNA cap structure plays an important role in translation initiation. Akichika et al. showed that m^6^Am does not modulate the binding affinity of eIF4E to the cap structure, suggesting that other cap-binding factors could be involved^7^. For instance, it has been shown that eIF3 and ABCF1 can selectively recognize 5′-UTR m^6^A and mediate the cap-independent translation^18,19^. Thus, future identification of potential m^6^Am readers, particularly those in the integrated stress response, could help elucidate the detailed function and mechanism of m^6^Am.

In summary, we report a tool for transcriptome-wide mapping of m^6^Am and 5′-UTR m^6^A, and reveal the dynamic features of the two epitranscriptomic marks under stress conditions. We anticipate that m^6^Am-seq will lead to new territories of RNA biology and open opportunities in the field of “cap epitranscriptomics”.

## Methods

### Antibodies and cell culture

Rabbit anti-m^6^A antibody was purchased from EMD Millipore (ABE572). Anti-m^7^G-cap monoclonal antibody was obtained from MBL (RN016M). HEK293T cells were cultured at 37 °C in DMEM medium supplemented with 10% FBS, 1% GlutaMAX and 0.5% penicillin/streptomycin with 5% CO_2_. For heat shock, cells were grown to a confluence of 80% in 10-cm plastic dishes, placed in a 43 °C water bath for 2 h, and then recover for 6 h in normal growing conditions. For hypoxia treatment, cells were grown to 80%, carried out in Hypoxia Hood which was maintained at 37 °C, 5% CO_2_, 1% oxygen for 24 h.

### RNA isolation

TRIzol was used for total RNA isolation according to the manufacturer’s protocol. <200 nt RNA was deleted using the MEGAclear Transcription Clean-Up kit (Ambion, AM1908).

### Quantification of m^6^A and m^6^Am by LC–MS/MS

For m^6^Am, 150 ng RNA was decapped by 10 units of RppH (NEB, M0356S) in Thermopol buffer for 5 h at 37 °C. RNA was purified by RNA clean & concentrator kit (Zymo Research, R1015). Purified RNA was digested by nuclease P1 (Sigma, N8630) with 10 mM ammonium acetate at 42 °C for 6 h, then mixed with 50 mM MES buffer (pH 6.5) and 0.5 U Shrimp Alkaline Phosphatase (NEB, M0371S), and incubated at 37 °C for another 6 h. For m^6^A, 150 ng RNA was digested into single nucleosides by nuclease P1 with 10 mM ammonium acetate at 42 °C for 6 h, then incubated with 50 mM MES buffer (pH 6.5) and 0.5 U Shrimp Alkaline Phosphatase for another 6 h at 37 °C.

The digested nucleosides were separated by the ultra-performance liquid chromatography on C18 column, and detected by the triple-quadrupole mass spectrometer (AB SCIEX QTRAP 6500). The multiple reaction-monitoring (MRM) mode was monitored: *m*/*z* 268.0–136.0 (A), *m*/*z* 245.0–113.1 (U), *m*/*z* 282.0–150.1 (m^6^A), *m*/*z* 296.0–150.0 (m^6^Am). The concentrations of m^6^A and m^6^Am levels in RNA samples were calculated from the standard curves.

### Synthesis of m^6^Am/Am and m^6^A probes

cap-m^6^Am/Am RNA: template was obtained by PCR, which contains the T7 promoter sequence and ~150 bp DNA sequence. Primers used to amplify the spike-in template was provided (Supplementary Data 6). In vitro transcription reaction contained: reaction buffer (ThermoFisher Scientific, AM1354), 800 ng template DNA, T7 Enzyme Mix (ThermoFisher Scientific, AM1354), 15 mM ATP/UTP/CTP/GTP, and 15 mM cap analogs (Trilink, N-7113 or N-7102, N-7102 was ordered via email addressed to orders@trilinkbiotech.com). The reaction was incubated overnight at 37 °C. After incubation, 1 μl of TURBO DNase (ThermoFisher Scientific, AM1354) was added to the reaction, and the reaction was incubated at 37 °C for 1 h. The RNA was purified by phenol–chloroform isolation and ethanol precipitation. The precise RNA was purified from 7 M urea/15% acrylamide gels using elution buffer (0.3 M sodium acetate, 1 mM EDTA, 0.05% SDS) at 37 °C for 12 h. Next, the RNA was precipitated with ethanol. The probes were used as spike-ins and for probe demethylation assay. The sequence of cap-m^6^Am/Am RNA probes was list as follows:

m^6^Am(Am)GGAGAAAAAUCACUCAGGGUCAAUGCCAGCGCUUCGUUAAUACAGAUGUAGGUGUUCCACAGGGUAGCCAGCAGCAUCCUGCGAUGCAGAUCCGGAACAUAAUGGUGCAGGGCGCUGACUUCCGCGUUUCCAGACUUUACGAAACACGGA.

The sequence of m^6^A probe was list as follows:

AUCUACCUGUCCAGUAGCCUUCAGGAUCAUGCUGUCUGACUUGCUGGm^6^ACAUCAUUCUAGUGCCAUAACUUCAGC.

### Synthesize the dually modified m^6^Am&m^6^A probe

The dually modified m^6^Am & m^6^A probe was obtained by utilizing T4 RNA ligase 2 to ligate the 3′ end of RNA probe to the 5′ end of another RNA probe. The 30nt for m^6^Am probe was obtained by in vitro transcription reaction. For phosphorylation of 5′end of m^6^A probe, RNA was treated by PNK (NEB, M0201S) with incubation for 1 h at 37 °C. 1 μl of 20 μM m^6^Am probe, 2 μl of 20 μM m^6^A probe, 1.5 μl of 20 μM splint DNA (CTACTGGACAGGTAGATAGTCTTGAAGGATTC), 1 μl 10× T4 DNA ligase buffer and 3 μl nuclease-free water were heated at 65 °C for 3 min, followed by 5 min at 25 °C in a thermocycler. Add 1 μl of 10 units/μl T4 RNA ligase 2 (NEB, M0239S) and 0.5 μl RiboLock RNase inhibitor (ThermoFisher Scientific, EO0381) into the reaction mixture and incubate at 37 °C for 2 h. Synthetic m^6^Am & m^6^A probe was gel purified and sliced in the desired size. The sequence of the m^6^Am & m^6^A probe was listed as follows:

Capm^6^AmGGAUAGCAGGCAUGGAAUCCUUCAAGACUAUCUACCUGUCCAGUAGCCUUCAGGAUCAUGCUGUCUGACUUGCUGGm^6^ACAUCAUUCUAGUGCCAUAACUUCAGC.

### In vitro probe assay

To investigate the FTO and ALKBH5 demethylation activity for m^6^Am and m^6^A, we performed in vitro demethylation assay using purified demethylases and synthesized probes contained m^6^A and m^6^Am. The demethylation assay was performed in 20 μl of mixture containing 200 ng probe, 50 mM MES (pH 6.5), 100 mM KCl, 2 mM MgCl_2_, 100 μM (NH_4_)_2_Fe(SO_4_)_2_·6H_2_O, 300 μM 2-ketoglutarate, 0 or 2 mM l-ascorbic acid, 0.4 U/ml SUPERase·In RNase inhibitor (Invitrogen, AM2694) and 2 μM purified FTO. The reaction mixture was incubated at 37 °C for 20 min. As the control, inactive demethylases were using for incubation with RNA.

### CAGE-seq library preparation

CAGE allows mapping of the transcriptional start sites of capped RNAs. 5 μg of total RNA purified from HEK293T cells was used for the CAGE-seq^45^. Reverse transcription was carried out using PrimeScript reverse transcriptase (Takara, 2680A) with the RT-N15-EcoP primer. The RNA-cDNA hybrid was diol oxidized with NaIO_4_ and then the 5′ Cap and 3′ end of RNA were biotinylated. The noncapped biotinylated RNA-cDNA hybrid was eliminated by RNase digestion and the biotin-trapping step. The complete cDNA was released from RNA and then the Bar-coded 5′ linker was ligated to the single-stranded cDNA. The second-strand cDNA was synthesized using the biotin-modified primer. The cDNA was digested with EcoP15I (NEB, R0646S) and the 3′ linker was ligated to the 3′ end of cDNA. The cDNA was performed PCR amplification. The CAGE library was sequenced on Illumina HiSeq 2000. The sequences of primers and linkers were provided (Supplementary Data 6).

### CAGE data analysis

CAGE tags were mapped to the human genome (hg19, UCSC Genome Browser) using TopHat2 (version 2.0.13) with default parameters that allow 0 mismatches per seed (22 nt), and CTSS pipeline was used to identify CAGE TSS^45^. Only uniquely mapped reads were used in downstream analysis within R and custom scripts. The mapped reads were sorted and the TSS tag was calculated. All unique 5′ ends of CAGE tag-supported TSS and the number of tags in each CTSS represents expression levels. Tag counts in each CTSS were normalized using tag pre million reads (TPMs). We also used Deeptools (version 3.5.0) to analyze the enrichment of m^6^Am in the −2 Kb to +2 Kb region around the CAGE TSS. The miCLIP-2015 m^6^Am list and miCLIP-2019 m^6^Am list were obtained from previous studies^10,13^.

### Cloning, expression, and purification of FTO and ALKBH5

Wild-type human FTO gene and ALKBH5 gene with deletion of the 66 amino acids were cloned into pET-28a. The recombinant plasmids were then transduced into *E. coli* BL21 (DE3). FTO and ALKBH5 proteins were purified following the procedures as previously described^46^. Bacteria were grown at 37 °C until the OD_600_ reached 0.8–1, and then induced with 0.5 mM IPTG overnight at 15 °C. Cells were harvested by centrifugation and then resuspended in ice-cold lysis buffer (25 mM Tris-HCl pH 8.0, 150 mM NaCl, 10 mM imidazole), disrupted on ice, and centrifuged at 11,000×*g* for 1 h at 4 °C. The supernatant was purified on HiTrap His column (GE Healthcare) and eluted with buffer containing 25 mM Tris-HCl pH 8.0, 150 mM NaCl, 500 mM imidazole. Next, the fractions were purified by HiTrap Q column (GE Healthcare). Finally, the fractions were purified by Gel-filtration chromatography (HiLoad 16/600 Superdex 200 pg column, GE Healthcare) equilibrated in storage buffer containing 25 mM Tris-HCl pH 8.0, 150 mM NaCl, and 3 mM dithiothreitol (DTT). Purified protein was stored at −80 °C with the addition of 20% glycerol.

### m^6^Am-seq

#### cap-m^7^G RNA immunoprecipitation

The total RNAs were extracted from control, heat shock-treated, and hypoxia-treated cells. 100 μg >200 nt RNA was fragmented into ~150 nt-long fragments using RNA fragmentation buffer (NEB, E6150S). Adding fragment stop solution buffer stopped the reaction, and fragments were purified and concentrated by ethanol precipitation. 10 ng of fragments were used as “input” and the remaining fragments were used for followed treatments. The fragmented RNA was denatured at 65 °C for 5 min, followed by chilling in ice. The denatured RNA was incubated overnight at 4 °C with 2 μg cap-m^7^G antibody (1:100 dilution) and 5 μl RiboLock RNase inhibitor in IPP buffer (10 mM Tris-HCl, pH 7.4, 150 mM NaCl, 0.1% NP-40). 20 μl of protein A/G UltraLink Resin (ThermoFisher Scientific, 53132) were washed twice with IPP buffer, and then resuspended in a 200 μl IPP buffer contained 5 μl RiboLock RNase inhibitor. The antibody-RNA mixture was then incubated with protein A/G UltraLink Resin at 4 °C for 3 h. The Resin was washed twice with 1 ml of IPP buffer, once with 1 ml of low-salt IP buffer (10 mM Tris-HCl, pH 7.4, 75 mM NaCl, 0.05% NP-40), once with 1 ml of high-salt IP buffer (10 mM Tris-HCl, pH 7.4, 200 mM NaCl, 0.05% NP-40) and twice with 1 ml TET buffer (10 mM Tris-HCl, pH 7.4, 1 mM EDTA, 0.05% NP-40) at 4 °C. The beads-antibody-RNA was resuspended in 1 ml Trizol and rotated for 10 min at room temperature. cap-m^7^G-containing RNA fragments were extracted and precipitated. Around 150 ng cap-m^7^G-immunoprecipitated RNA fragments could be obtained.

#### In vitro demethylation treatment

1 ng of the cap-m^7^G-immunoprecipitated RNA was used as “m^7^G-IP”. RNA was subjected to [FTO (+)] or [FTO (−)] treatment. About 100 ng of RNA was used for [FTO (+)] treatment. RNA was denatured at 65 °C for 5 min, followed by chilling in ice. And then, the demethylation assay was performed in a 20 μl mixture which contained 50 mM MES (pH 6.5), 100 mM KCl, 2 mM MgCl_2_, 100 μM (NH_4_)_2_Fe(SO_4_)_2_·6H_2_O, 300 μM 2-ketoglutarate, 0.4 U/ml SUPERase·In RNase inhibitor and 1 μM purified FTO. After incubation at 37 °C for 20 min, the demethylated RNA was purified by phenol–chloroform extraction. The remaining RNA (around 50 ng) directly performed m^6^A RNA immunoprecipitation.

#### m^6^A RNA immunoprecipitation

The [FTO (−)] and [FTO (+)] samples were immunoprecipitated with m^6^A antibody. The m^6^A RNA immunoprecipitation was performed following the procedures as previously described^21^. 10 μl of protein A magnetic beads (ThermoFisher Scientific, 10002D) and 10 μl of protein G magnetic beads (ThermoFisher Scientific, 10004D) were mixed and washed twice with 500 μl of IPP buffer (10 mM Tris-HCl, pH 7.4,150 mM NaCl, 0.1% NP-40). The beads were resuspended in 500 μl of IPP buffer containing 2 μg of anti-m^6^A antibody (Millipore, ABE572). The mixture was incubated at 4 °C for at least 6 h. The beads-antibody mixture was washed twice in 500 μl of IPP buffer and resuspended with 200 μl mixture containing 40 μl of 5× IPP buffer, denatured RNA (the [FTO (−)] control and [FTO (+)] treated samples) and 5 μl of RNasin Plus RNase Inhibitor (Promega, N2615) at 4 °C for 2 h (1:100 dilution of anti-m^6^A antibody). The beads-antibody-RNA mixture was washed twice with 500 μl of IPP buffer, twice with 500 μl of low-salt IP buffer (10 mM Tris-HCl, pH 7.5, 50 mM NaCl, 0.1% NP-40), and twice with 500 μl of high-salt IP buffer (10 mM Tris-HCl, pH 7.5, 500 mM NaCl,0.1% NP-40). And then, bound fragments were eluted from beads with 200 μl of IPP buffer containing 6.7 mM N^6^-methyladenosine (Sigma-Aldrich, M2780) and 5 μl of RiboLock RNase inhibitor. An additional phenol–chloroform isolation and ethanol precipitation were used to purify the RNA. The bound RNA was called [FTO (+) m^6^A-IP] and [FTO (−) m^6^A-IP].

#### Library preparation and sequencing

The “input”, “m^7^G-IP”, [FTO (+) m^6^A-IP] and [FTO (−) m^6^A-IP] were subjected to library construction using SMARTer® Stranded Total RNA-Seq Kit v2—Pico Input Mammalian (634413, Takara – Clontech, Japan) according to the manufacturer’s protocol. The libraries were sequenced on Illumina HiSeq X10 with PE150.

### Reads pre-processing and alignment

Strand orientation of the original RNA was preserved on the process of library construction and reads R2 yields sequences sense to the original RNA. Thus, only reads R2 was used in our study. Raw sequencing reads were firstly subjected to Trim_galore (version 0.6.6, http://www.bioinformatics.babraham.ac.uk/projects/trim_galore/) and cutadapt software (version 1.18) for quality control and trimming adaptor. The quality threshold was set to 30, and the minimum length required for reads after trimming was 20 nt. Processed reads were mapped to genome (hg19, UCSC Genome Browser) using HISAT2 (version 2.1.0)^47^ with default parameters, separated by strand with in-house scripts, and reads coverage were showed by IGV. The mpileup files are generated from mapped BAM files using samtools mpileup command (version 1.9).

### Transcriptome-wide identification of 5′-UTR peaks in m^6^A-IP sample

Mapped reads were subjected to the RNA methylation peak caller exomePeak^48^, the FOLD_ENRICHMENT was set to 3, the GENE_ANNO_GTF were set RefSeq_hg19 for peak calling. For genome-base peak caller MACS2 (version 2.1.1)^49^, the effective genome size was set to 2.7*10^9^ for human, under the option of *-nomodel* and *q*-value cutoff 0.01. In order to identify high-confidence peak in m^6^A-IP sample, peak must meet the following conditions:(1) peak was overlapped between m^6^A-IP and m^7^G-IP samples, and (2) fold enrichment in m^6^A-IP higher than m^7^G-IP samples. Only peaks that can meet both requirements were used for m^6^A/m^6^Am signal identification in our study.

### Definition and calculation of the demethylase-sensitivity peaks

In order to more accurately extract the m^6^Am peak and 5′-UTR m^6^A peak, we identify the demethylase-sensitivity peak between the FTO (+) and FTO (−) sample. Firstly, 5′-UTR peak defined in the previous section of “Transcriptome-wide identification of 5′-UTR peaks in m^6^A-IP sample” were used for analysis. Secondly, differential methylation peak between FTO (+) and FTO (−) samples were identified using exomePeak (*P* < 0.01) and DESeq2 (version 3.11)^50^ in the R environment (version 3.6). Thirdly, when the ratio of FTO (−) to FTO (+) is >2, is defined as demethylase-sensitivity peak.

### Identification of m^6^Am and 5′-UTR m^6^A peaks

An m^6^Am peak was identified when: (1) peak was high-confidence in m^6^A-IP sample; (2) peak was demethylase sensitivity between the FTO (+) and FTO (−) sample. The 5′-UTR m^6^A peak was identified when: (1) peak was high-confidence in m^6^A-IP sample; (2) peak was demethylase insensitivity between the FTO (+) and FTO (−) sample, and was not decrease after FTO treatment.

### Identification of m^6^Am site

To identify the potential m^6^Am site, we defined a “start rate difference” score (SRD score) for each nucleotide within an m^6^Am peak. SRD score takes into account normalized sequencing start rate in untreated samples m1 = [FTO (−) start reads]/[FTO (−) depth] and the reads coverage difference within the FTO (+) and FTO (−) samples m2 = [FTO (−) depth − FTO (+) depth]/[FTO (−) depth]. m1 reflects the fact that m^6^Am site is the first transcribed nucleotide, while m2 is a measure for demethylase sensitivity. Because the start rate in FTO (+) samples could be very low and hence prone to the high background, we used FTO (+) coverage in m2 instead of FTO (+) start rate.

1 $${{{\rm{SRD}}}}\,{{{\rm{score}}}}={{{\rm{m1}}}}+{{{\rm{m2}}}}$$

The SRD score was used to identify the m^6^Am site. The defined a position *i* was defined to be m^6^Am when the following criteria were met: (1) position *i* must be located within m^6^Am peak and carry adenosine residue; (2) the start reads of position *i* are not <20 in FTO (−) sample; (3) the start rate of position i in FTO (−) sample is greater than that in m^7^G-IP sample; (4) the start reads coverage in m^7^G-IP sample is greater than that in input; (5) the position i possesses the top 3 of SRD scores (SRD score > 1).

### Identification of stress-induced peak

In order to identify the stress-induced peak, differential methylation peaks between the untreated and stress sample were calculate using exomePeak (version 2.13, *P* < 0.01) and DESeq2. The stress-induced peak was defined, when the difference between the stress and untreated sample is >2.

### Identification of PCIF1-dependent peak

The m^6^Am peak in WT and PCIF1-KO sample was calculated in section “Identification of m^6^Am and 5′UTR m^6^A peak”. To identification, the PCIF1-dependent peak, differential methylation peaks between WT and PCIF1-KO samples were calculate using exomePeak and DESeq2. The PCIF1-dependent peak was extracted, when the differential methylation ratio of WT to PCIF1-KO sample is >2 and *P*-value < 0.05.

### Analysis of RNA-seq data

Paired-end, adapter-clean reads were aligned to the human genome (hg19, UCSC Genome Browser) using TopHat2 (version 2.0.13) with default parameters. The gene expression level was quantified as FPKM by Cufflinks (version 2.2.1)^51^.

### Correlations analysis of m^6^Am site with histone modification and TSS

H3K27ac, H3K4me3, H3K4me1 and H3K9me3 ChIP-seq data was downloaded from ENCODE portal^52^ (https://www.encodeproject.org/) with the following identifiers: ENCSR000DTU, ENCSR000FCJ, ENCSR000FCH, ENCSR000FCG. GRO-seq data were downloaded from the GEO database (GSE92375)^53^. We divided the m^6^Am sites into two parts with and without BCA motif, m^6^Am sites was extended 500 bp for each site, the overlapped site was removed. Deeptools were used to analyze ChIP-seq enrichment in −5 Kb to +5 Kb region around the m^6^Am sites.

### Motif discovery and GO enrichment analysis

Peaks were annotated by homer software (version 4.10) with hg19 reference genome to annotate the marked genes. For the analysis of sequence consensus, the top 1000 peaks were chosen for de novo motif analysis with MEME (version 4.12.0)^54^, with 100 nt-long peak summit centered sequences as input. Gene Ontology (GO) enrichment analyses were performed using DAVID web-based tool (version 6.8)^55^.

### Statistical analysis and quantification

For the statistical analysis of results, statistical evaluation was performed by unpaired two-sided Mann–Whitney *U*-test.

### Reporting summary

Further information on research design is available in the Nature Research Reporting Summary linked to this article.

## Supplementary information

Supplementary Information

Reporting Summary

Supplementary Data 1

Supplementary Data 2

Supplementary Data 3

Supplementary Data 4

Supplementary Data 5

Supplementary Data 6

Description of Additional Supplementary Files

## Data Availability

The sequence data generated in this study have been deposited in the NCBI GEO, under accession code GSE180253 that is publicly accessible at https://www.ncbi.nlm.nih.gov/geo/query/acc.cgi?acc=GSE180253. For H3K27ac, H3K4me3, H3K4me1, and H3K9me3 ChIP-seq public data were downloaded from ENCODE portal^52^ (https://www.encodeproject.org/) with the following identifiers: ENCSR000DTU, ENCSR000FCJ, ENCSR000FCH, and ENCSR000FCG. GRO-seq data were downloaded from the GEO database (GSE92375)^53^. Source data are provided with this paper.

## Source data

Source Data

## Code availability

Custom code used for m^6^Am-seq analysis is available on GitHub (https://github.com/KaiLi-bioinfo/m6Am_seq/).
