# Supplementary material for: m6Am-seq reveals the dynamic m6Am methylation in the human transcriptome
Source: Nat Commun. 2021 Aug 6;12:4778. doi: 10.1038/s41467-021-25105-5 (PMC8346571; doi:10.1038/s41467-021-25105-5)
Supplement: Supplementary file 1 — Supplementary Information [file 41467_2021_25105_MOESM1_ESM.pdf]

m^6Am-seq reveals the dynamic m^6Am methylation in the human transcriptome

Hanxiao Sun[1,5], Kai Li[1,2,3,5], Xiaoting Zhang[1], Jun'e Liu[1], Meiling Zhang[1], Haowei Meng[1] & Chengqi Yi[1,3,4,*]

[1]*State Key Laboratory of Protein and Plant Gene Research, School of Life Sciences, Peking University, Beijing 100871, China.*

[2]*Academy for Advanced Interdisciplinary Studies, Peking University, Beijing 100871, China.*

[3]*Peking-Tsinghua Center for Life Sciences, Peking University, Beijing, China.*

[4]*Department of Chemical Biology and Synthetic and Functional Biomolecules Center, College of Chemistry and Molecular Engineering, Peking University, Beijing 100871, China.*

[5]*These authors contributed equally to this work.*

[*]*Correspondence: chengqi.yi@pku.edu.cn (C. Y.)*

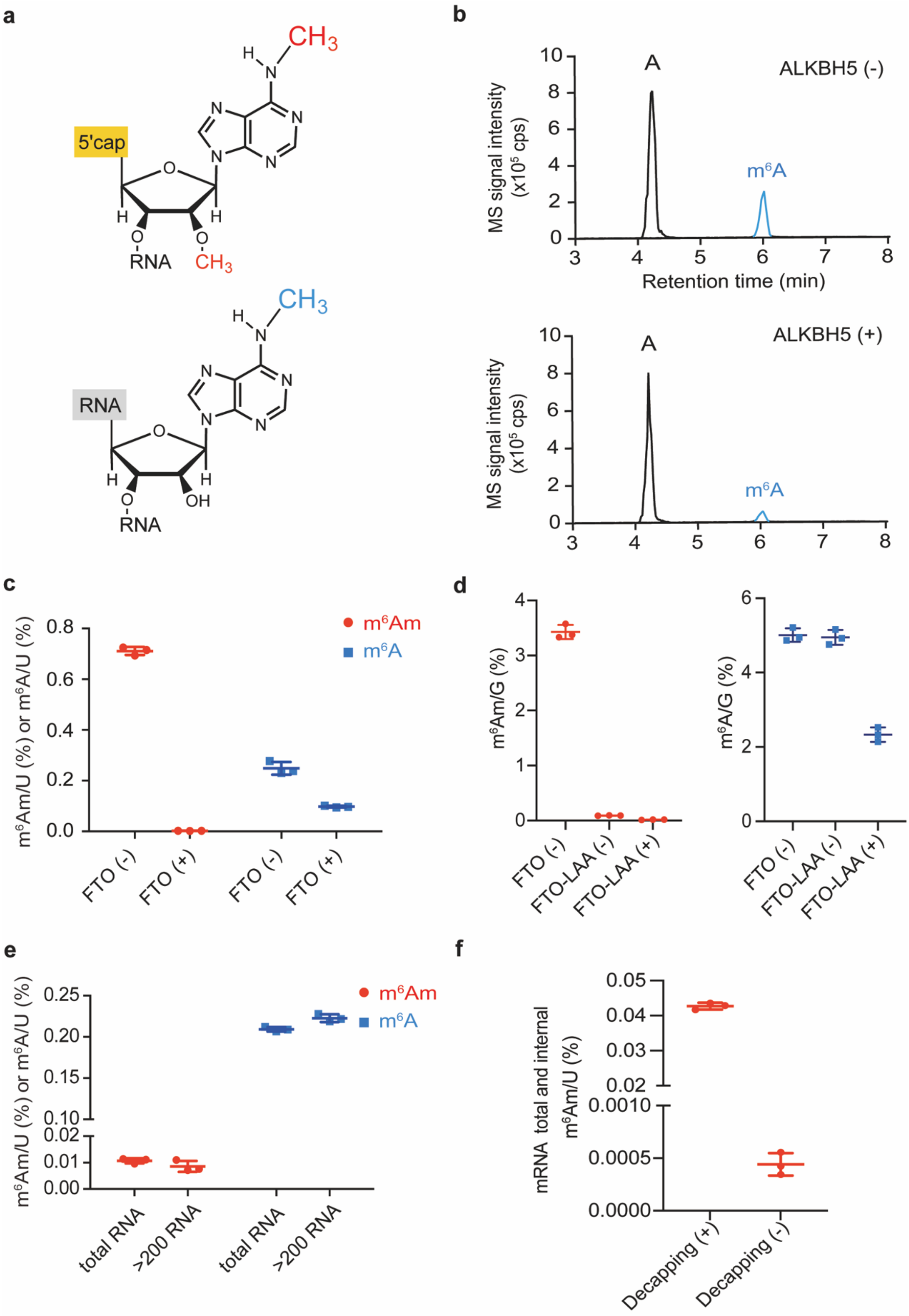

a
5'cap
RNA
b
ALKBH5 (-)
ALKBH5 (+)
MS signal intensity (x10^5 cps)
Retention time (min)
A
m6A
c
m6Am
m6A
m6Am/U (%) or m6A/U (%)
FTO (-)
FTO (+)
d
m6Am/G (%)
m6A/G (%)
FTO-LAA (-)
FTO-LAA (+)
e
total RNA
>200 RNA
f
mRNA total and internal m6Am/U (%)
Decapping (+)
Decapping (-)

Supplementary Figure 1. *In vitro* demethylation activity of FTO and ALKBH5. (**a**) Chemical structures and sequence contexts of m^6Am and m^6A. (**b**) Demethylation of m^6A by recombinant ALKBH5. ~80% m^6A was removed. ALKBH5 (-) denotes that inactivated ALKBH5 was used as a control. (**c**) Demethylation of m^6Am and m^6A in synthesized RNA probes by recombinant FTO under literature recommended conditions. >98% m^6Am and >60% m^6A could be demethylated. FTO (-) denotes that inactivated FTO was used as a control. Values represent mean ± SD (*n* = 3 independent samples for each group). Source data are provided as a Source Data file. (**d**) Selectivity of FTO-mediated demethylation assessed on a m^6Am & m^6A dually modified RNA probe. The FTO demethylation efficiency on m^6Am and m^6A modifications. FTO (-) denotes that inactivated FTO was used as a control. FTO-LAA (-) or (+) denotes that LAA is absent or present in the demethylation condition. Values represent mean ± SD (*n* = 3 independent samples for each group). Source data are provided as a Source Data file. (**e**) Quantitative LC-MS/MS analysis of m^6Am and m^6A levels in total RNA and >200nt total RNA from HEK293T cells. Values represent mean ± SD (*n* = 3 independent samples for each group). Source data are provided as a Source Data file. (**f**) The levels of total and potential internal m^6Am modification in polyadenylated RNAs. Values represent mean ± SD (*n* = 3 independent samples for each group). Source data are provided as a Source Data file.

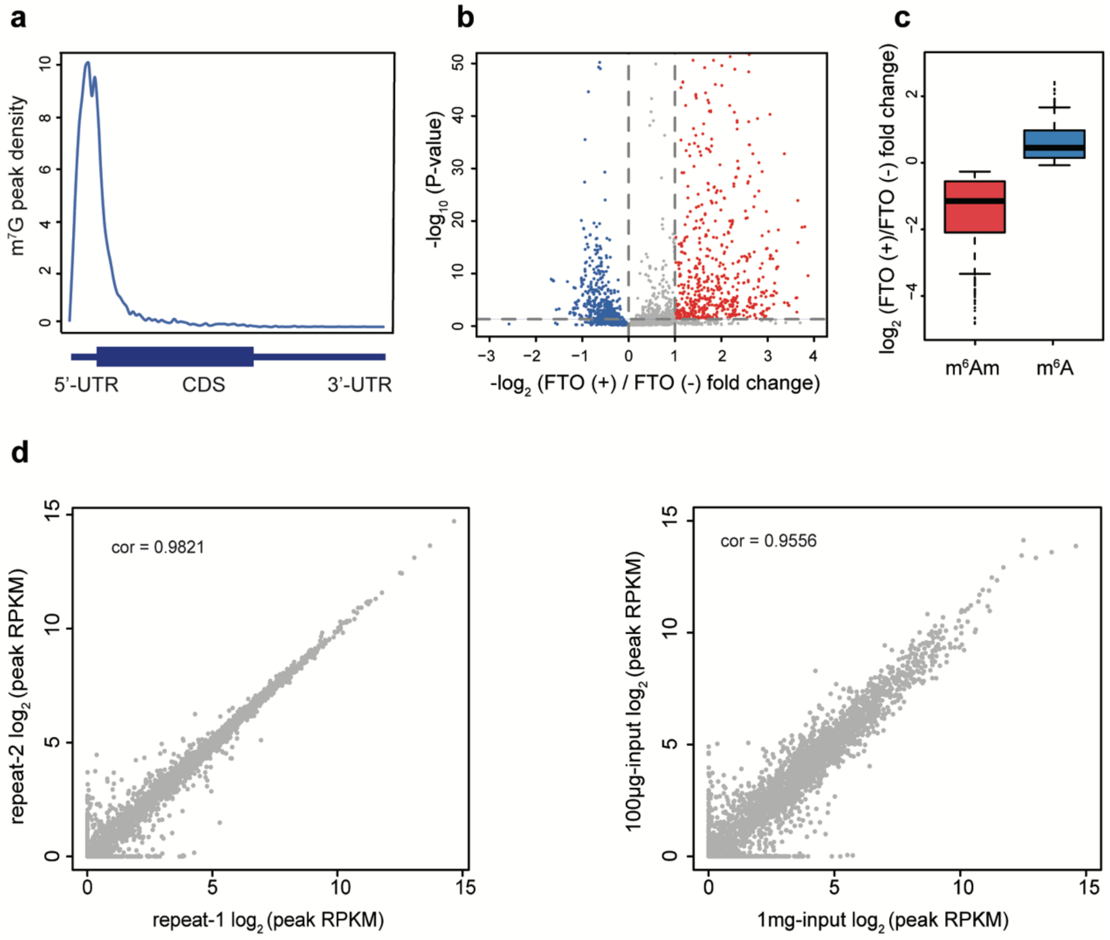

Supplementary Figure 2. m^{6}Am-seq involves two IP steps for m^{6}Am and 5'-UTR m^{6}A detection. (**a**) Metagene profiles of m^{7}G peak distribution across mRNA segments. Source data are provided as a Source Data file. (**b**) Volcano plots depicting the bimodal-like distribution of "demethylase-sensitive" peaks (red bots) and "demethylase-insensitive" peaks (blue bots). (**c**) Boxplot showing that the fold change of peak intensity of m^{6}Am (red, n=1,652), but not that of m^{6}A (blue, n=1,307), was significantly reduced upon FTO demethylation treatment. Boxes represent 25th–75th percentile (line at median) with whiskers at 1.5*IQR. (**d**) Scatter plots showing RPKM values for the two biological replicates of m^{6}Am-seq (Pearson's r = 0.9821) and for two experiments with different amount of input samples (Pearson's r = 0.9556). Source data are provided as a Source Data file.

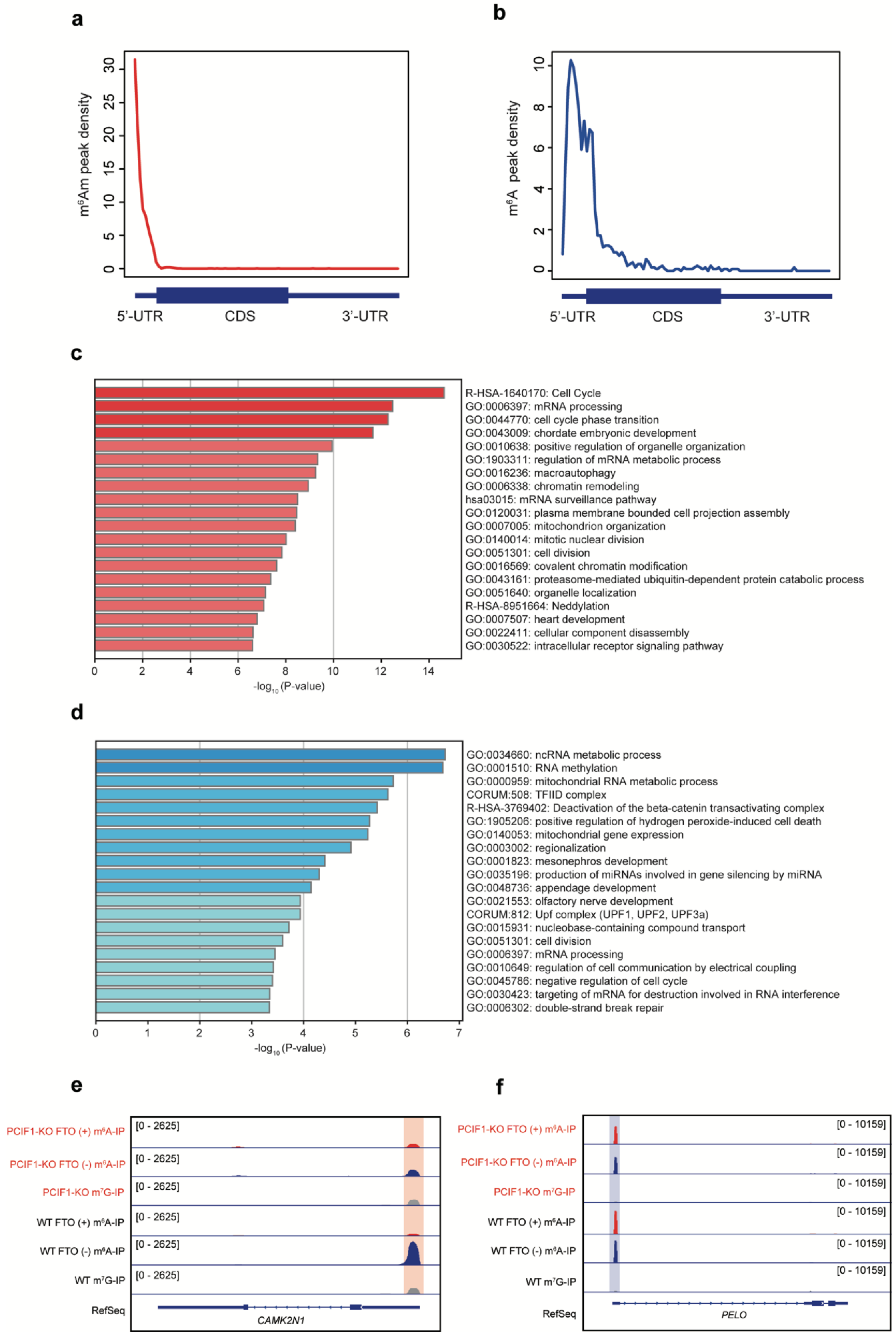

a
m^{6}Am peak density
0
5
10
15
20
25
30
5'-UTR
CDS
3'-UTR
b
m^{6}A peak density
0
2
4
6
8
10
5'-UTR
CDS
3'-UTR
c
R-HSA-1640170: Cell Cycle
GO:0006397: mRNA processing
GO:0044770: cell cycle phase transition
GO:0043009: chordate embryonic development
GO:0010638: positive regulation of organelle organization
GO:1903311: regulation of mRNA metabolic process
GO:0016236: macroautophagy
GO:0006338: chromatin remodeling
hsa03015: mRNA surveillance pathway
GO:0120031: plasma membrane bounded cell projection assembly
GO:0007005: mitochondrion organization
GO:0140014: mitotic nuclear division
GO:0051301: cell division
GO:0016569: covalent chromatin modification
GO:0043161: proteasome-mediated ubiquitin-dependent protein catabolic process
GO:0051640: organelle localization
R-HSA-8951664: Neddylation
GO:0007507: heart development
GO:0022411: cellular component disassembly
GO:0030522: intracellular receptor signaling pathway
0
2
4
6
8
10
12
14
-log$_{10}$ (P-value)
d
GO:0034660: ncRNA metabolic process
GO:0001510: RNA methylation
GO:0000959: mitochondrial RNA metabolic process
CORUM:508: TFIID complex
R-HSA-3769402: Deactivation of the beta-catenin transactivating complex
GO:1905206: positive regulation of hydrogen peroxide-induced cell death
GO:0140053: mitochondrial gene expression
GO:0003002: regionalization
GO:0001823: mesonephros development
GO:0035196: production of miRNAs involved in gene silencing by miRNA
GO:0048736: appendage development
GO:0021553: olfactory nerve development
CORUM:812: Upf complex (UPF1, UPF2, UPF3a)
GO:0015931: nucleobase-containing compound transport
GO:0051301: cell division
GO:0006397: mRNA processing
GO:0010649: regulation of cell communication by electrical coupling
GO:0045786: negative regulation of cell cycle
GO:0030423: targeting of mRNA for destruction involved in RNA interference
GO:0006302: double-strand break repair
0
1
2
3
4
5
6
7
-log$_{10}$ (P-value)
e
PCIF1-KO FTO (+) m^{6}A-IP
PCIF1-KO FTO (-) m^{6}A-IP
PCIF1-KO m^{7}G-IP
WT FTO (+) m^{6}A-IP
WT FTO (-) m^{6}A-IP
WT m^{7}G-IP
RefSeq
[0 - 2625]
CAMK2N1
f
PCIF1-KO FTO (+) m^{6}A-IP
PCIF1-KO FTO (-) m^{6}A-IP
PCIF1-KO m^{7}G-IP
WT FTO (+) m^{6}A-IP
WT FTO (-) m^{6}A-IP
WT m^{7}G-IP
RefSeq
[0 - 10159]
PELO

Supplementary Figure 3. m^{6}Am-seq reveals the distribution of m^{6}Am and 5'-UTR m^{6}A in the human transcriptome. Distribution of m^{6}Am peaks (**a**) and 5'-UTR m^{6}A (**b**) peaks along mRNA segments. Source data are provided as a Source Data file. GO analysis based on DAVID database of the high-confidence m^{6}Am (**c**) or 5'-UTR m^{6}A (**d**) containing transcripts (*P*-value provided by DAVID). (**e-f**) Depletion of PCIF1 further confirms m^{6}Am and 5'-UTR m^{6}A identified by m^{6}Am-seq. (**e**) An m^{6}Am peak identified by m^{6}Am-seq within 5'end of *CAMK2N1* was lost in the PCIF1 KO datasets, suggesting that this is a genuine m^{6}Am modification. (**f**) 5'-UTR m^{6}A in *PELO* identified by m^{6}Am-seq was not affected in the PCIF1 KO datasets, supporting it as a 5'-UTR m^{6}A peak. Pink and blue background colors denote m^{6}Am and m^{6}A signals, respectively.

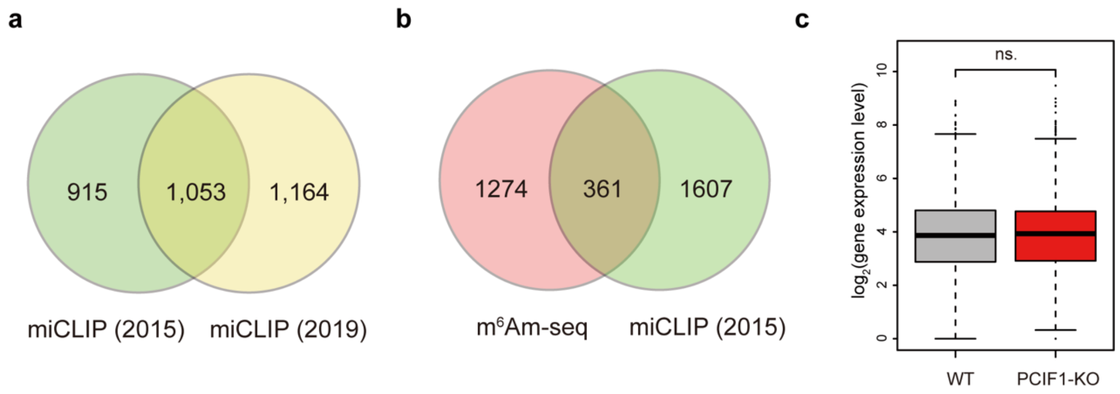

Supplementary Figure 4. Detailed comparison between m^{6}Am-seq and miCLIP. (**a**) Venn diagram showing the poor overlap of the m^{6}Am list reported in 2015 and that reported in 2019 by miCLIP. (**b**) Venn diagram showing the overlap of the m^{6}Am list reported in 2015 by miCLIP and the m^{6}Am list by m^{6}Am-seq. (**c**) The mRNA expression level of m^{6}Am marked transcripts didn't change upon PCIF1 KO (n=1,635). Statistical significance of the difference was determined by unpaired two-sided Mann-Whitney U-test. Boxes represent 25th–75th percentile (line at median) with whiskers at 1.5*IQR. Source data are provided as a Source Data file.

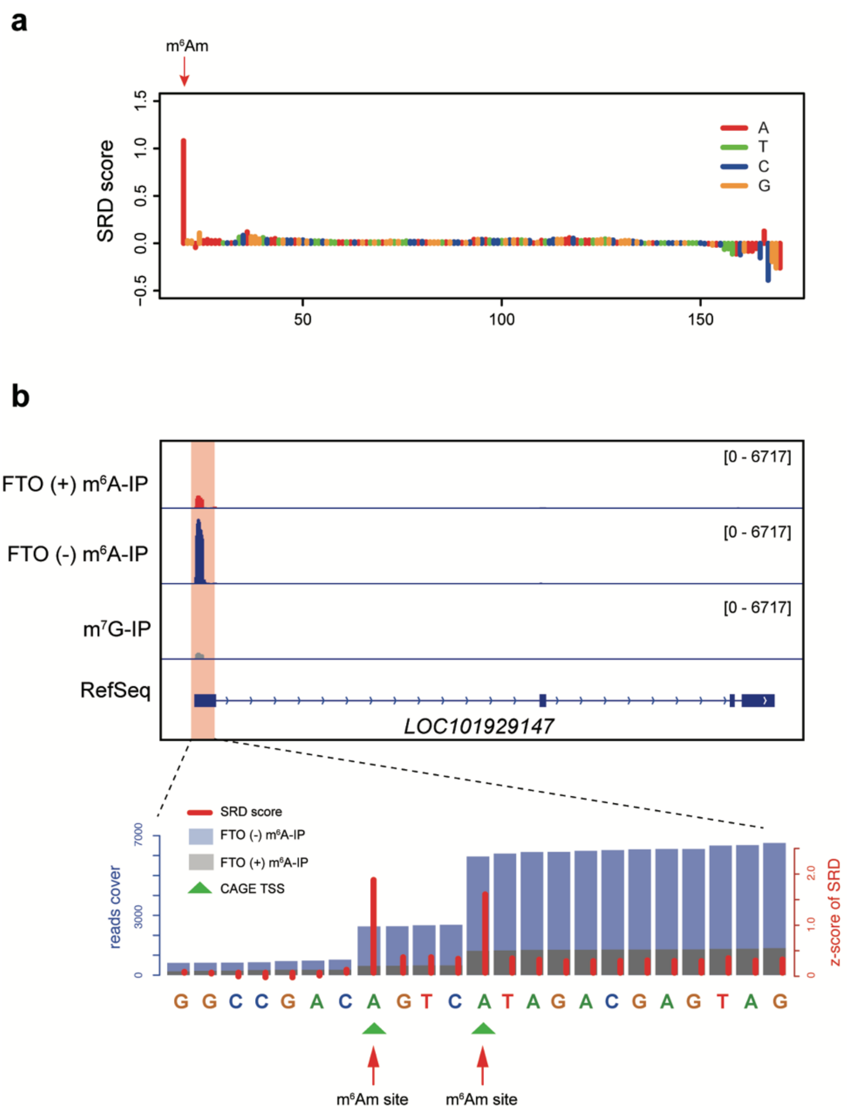

Supplementary Figure 5. m^{6}Am-seq identifies m^{6}Am at single-base resolution. (**a**) The m^{6}Am site in synthetic spike-in RNAs was actually identified. Source data are provided as a Source Data file. (**b**) A representative view of two single m^{6}Am sites on the transcripts of lncRNA *LOC101929147*, which were both supported by CAGE data. Two adenosine residues with high SRD score (red bar) were defined as m^{6}Am sites, which overlapped exactly with CAGE sites (green triangles). Each segment was normalized according to its average length in RefSeq annotation. Pink background color denotes m^{6}Am signal.

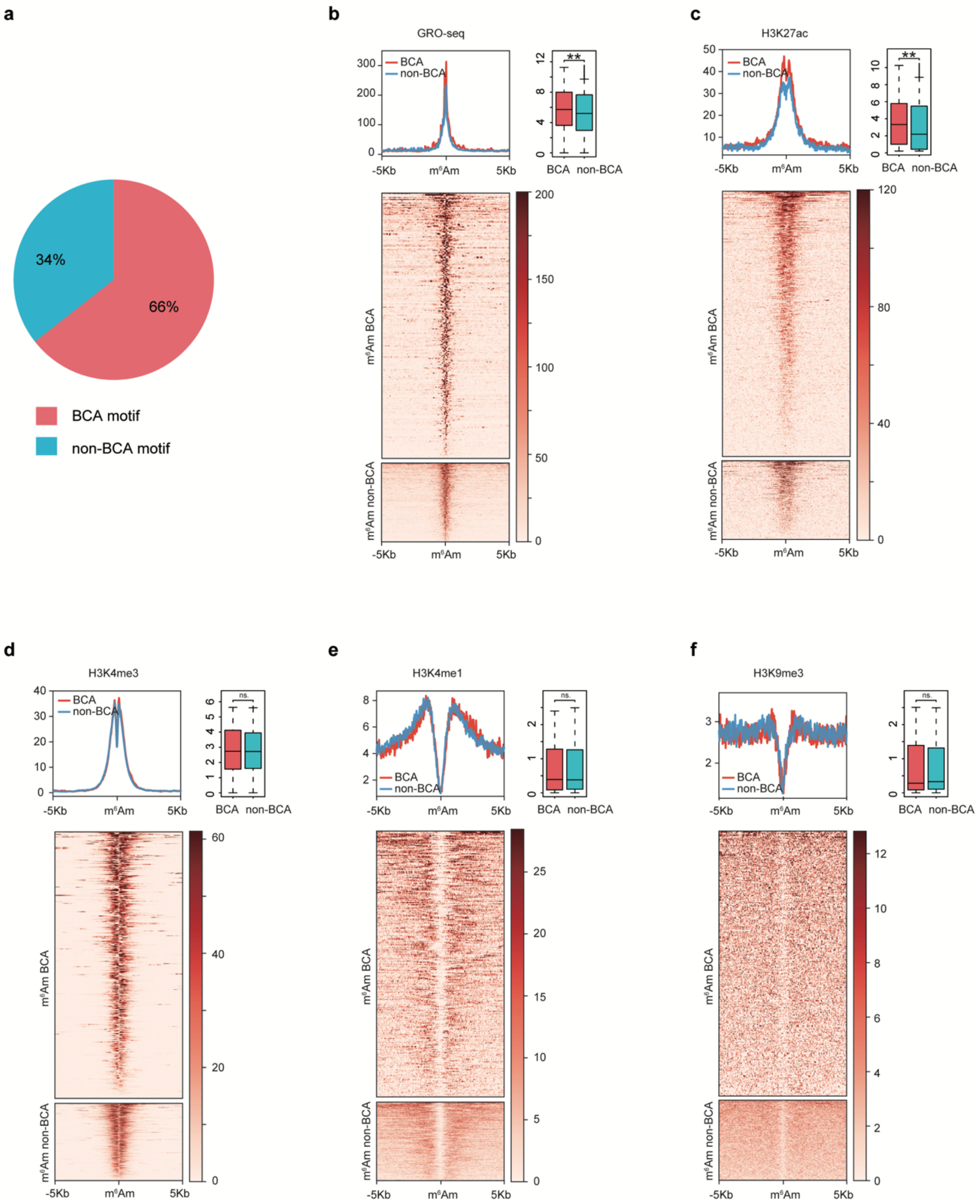
a
34%
66%
BCA motif
non-BCA motif
b
GRO-seq
BCA
non-BCA
-5Kb
m6Am
5Kb
m6Am BCA
m6Am non-BCA
c
H3K27ac
d
H3K4me3
ns.
e
H3K4me1
f
H3K9me3

Supplementary Figure 6. m^{6}Am sites with BCA-motif show stronger GRO-seq and H3K27ac signal. (**a**) The motif proportion of m^{6}Am sites. (**b-f**) The metaplot and boxplot showing the signal enrichment of GRO-seq, **P = 0.00237 (**b**), H3K27ac ChIP-seq, **P = 0.00382 (**c**), H3K4me3 ChIP-seq (**d**), H3K4me1 ChIP-seq (**e**) and H3K9me3 ChIP-seq (**f**) in -5Kb ~ +5Kb region around the m^{6}Am sites with BCA motif (red, n=1,090) or without BCA motif (blue, n=562). Statistical significance of the difference was determined by unpaired two-sided Mann-Whitney U-test. Boxes represent 25th–75th percentile (line at median) with whiskers at 1.5*IQR.

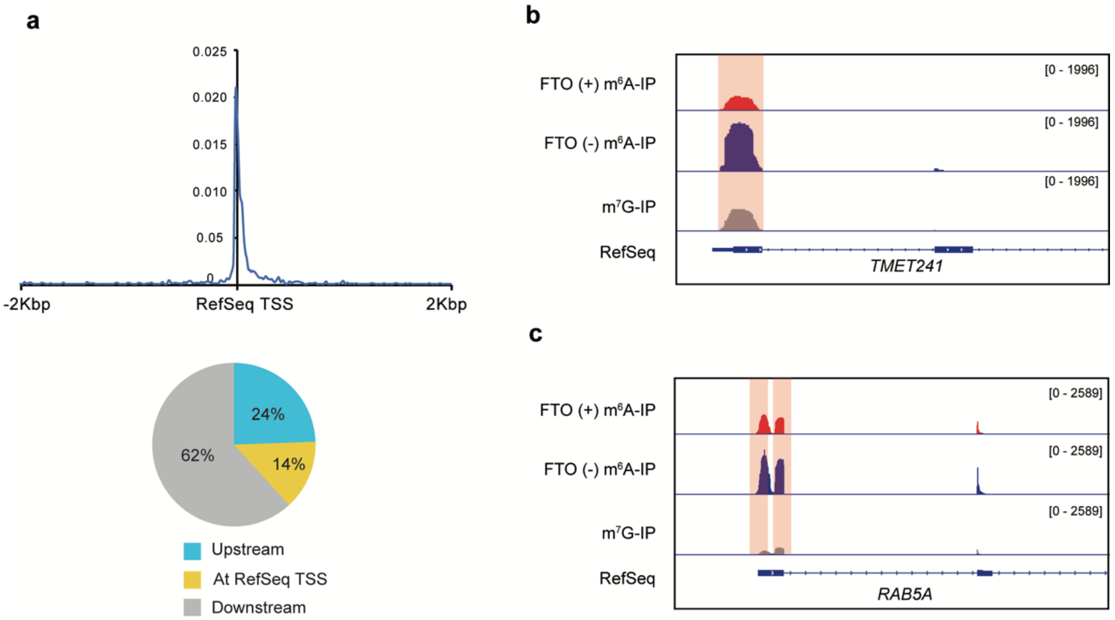

Supplementary Figure 7. m^{6}Am-seq reveals more accurate TSS information than annotated TSSs. (**a**) A metaplot showing the identified m^{6}Am sites with RefSeq-annotated TSSs. Source data are provided as a Source Data file. (**b**) Genome browser view showed that the m^{6}Am peak of *TMET241* would have been located within 5'-UTR, if annotated TSS produced by RefSeq was used. (**c**) Representative view of the m^{6}Am peaks on two isoforms of *RAB5A*. Pink background color denotes m^{6}Am signal.

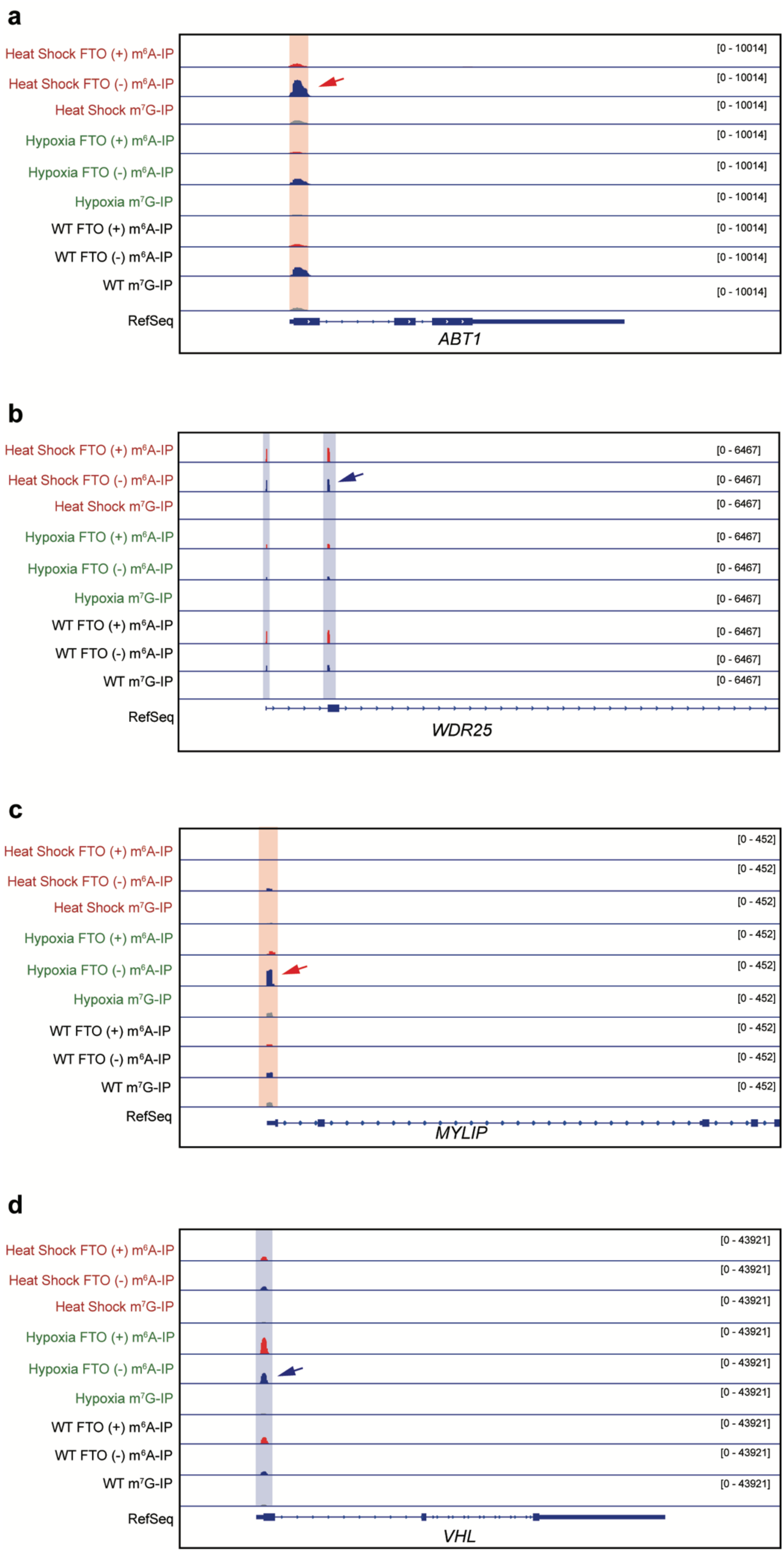
a
Heat Shock FTO (+) m6A-IP
Heat Shock FTO (-) m6A-IP
Heat Shock m7G-IP
Hypoxia FTO (+) m6A-IP
Hypoxia FTO (-) m6A-IP
Hypoxia m7G-IP
WT FTO (+) m6A-IP
WT FTO (-) m6A-IP
WT m7G-IP
RefSeq
[0 - 10014]
ABT1
b
Heat Shock FTO (+) m6A-IP
Heat Shock FTO (-) m6A-IP
Heat Shock m7G-IP
Hypoxia FTO (+) m6A-IP
Hypoxia FTO (-) m6A-IP
Hypoxia m7G-IP
WT FTO (+) m6A-IP
WT FTO (-) m6A-IP
WT m7G-IP
RefSeq
[0 - 6467]
WDR25
c
Heat Shock FTO (+) m6A-IP
Heat Shock FTO (-) m6A-IP
Heat Shock m7G-IP
Hypoxia FTO (+) m6A-IP
Hypoxia FTO (-) m6A-IP
Hypoxia m7G-IP
WT FTO (+) m6A-IP
WT FTO (-) m6A-IP
WT m7G-IP
RefSeq
[0 - 452]
MYLIP
d
Heat Shock FTO (+) m6A-IP
Heat Shock FTO (-) m6A-IP
Heat Shock m7G-IP
Hypoxia FTO (+) m6A-IP
Hypoxia FTO (-) m6A-IP
Hypoxia m7G-IP
WT FTO (+) m6A-IP
WT FTO (-) m6A-IP
WT m7G-IP
RefSeq
[0 - 43921]
VHL

Supplementary Figure 8. m^{6}Am and 5'-UTR m^{6}A is dynamically regulated by stress conditions. Representative views of heat shock-inducible m^{6}Am peaks (**a**) and 5'-UTR m^{6}A peaks (**b**) in human mRNA. Representative views of hypoxia-inducible m^{6}Am peaks (**c**) and 5'-UTR m^{6}A peaks (**d**) in human mRNA. Red arrows denote stress-inducible m^{6}Am peaks; blue arrows denote stress-inducible 5'-UTR m^{6}A peaks. Pink and blue background colors denote m^{6}Am and m^{6}A signals, respectively.

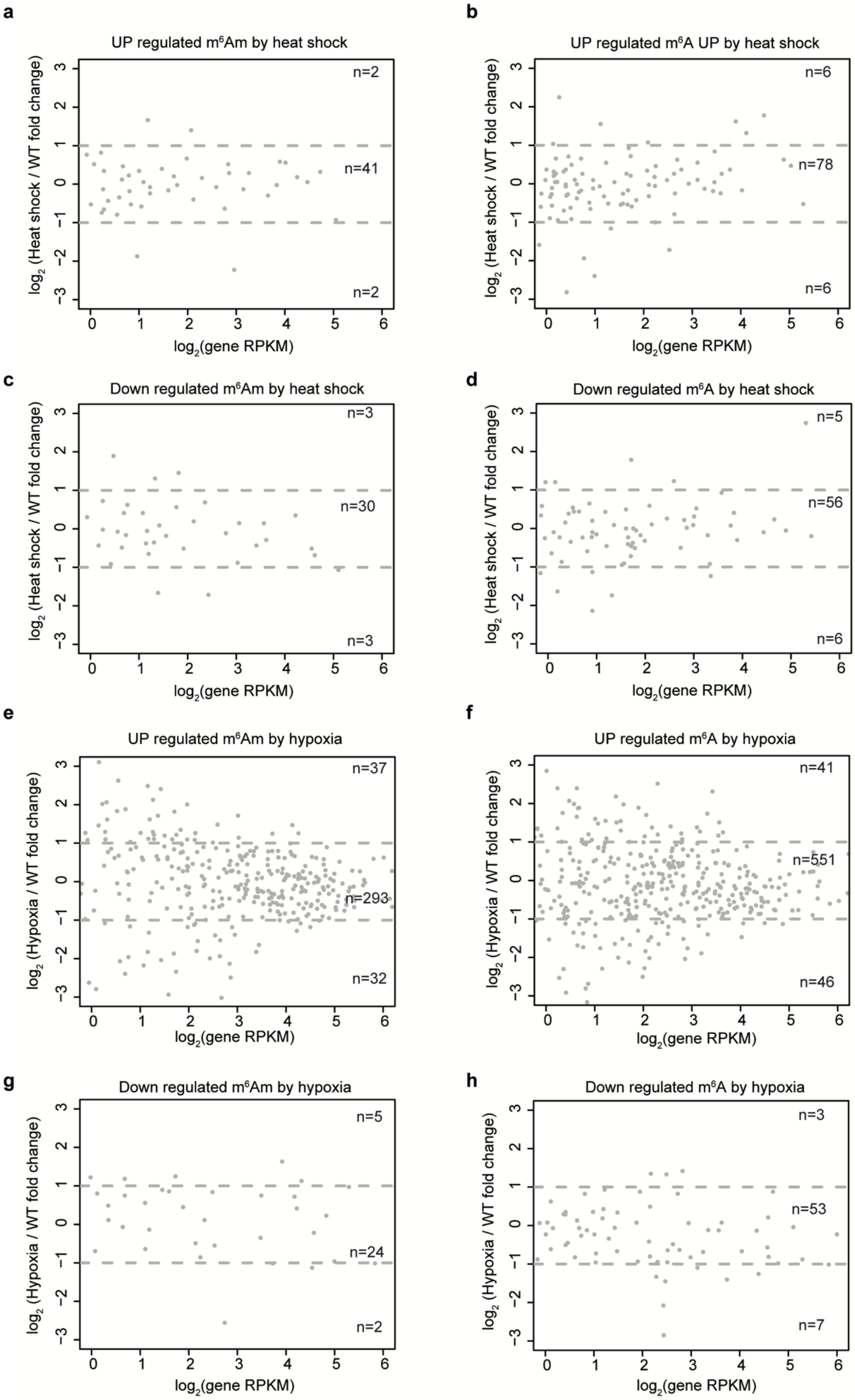
a
UP regulated m6Am by heat shock
n=2
n=41
n=2
log2 (Heat shock / WT fold change)
log2(gene RPKM)
b
UP regulated m6A UP by heat shock
n=6
n=78
n=6
log2 (Heat shock / WT fold change)
log2(gene RPKM)
c
Down regulated m6Am by heat shock
n=3
n=30
n=3
log2 (Heat shock / WT fold change)
log2(gene RPKM)
d
Down regulated m6A by heat shock
n=5
n=56
n=6
log2 (Heat shock / WT fold change)
log2(gene RPKM)
e
UP regulated m6Am by hypoxia
n=37
n=293
n=32
log2 (Hypoxia / WT fold change)
log2(gene RPKM)
f
UP regulated m6A by hypoxia
n=41
n=551
n=46
log2 (Hypoxia / WT fold change)
log2(gene RPKM)
g
Down regulated m6Am by hypoxia
n=5
n=24
n=2
log2 (Hypoxia / WT fold change)
log2(gene RPKM)
h
Down regulated m6A by hypoxia
n=3
n=53
n=7
log2 (Hypoxia / WT fold change)
log2(gene RPKM)

Supplementary Figure 9. The mRNA levels of most of genes containing stress-regulated modifications are unaltered. MA-plot showing fold change of mRNA expression after stress conditions for genes containing heat shock-induced m^6Am (**a, c**), heat shock-induced 5'-UTR m^6A (**b, d**), hypoxia-induced m^6Am (**e, g**) and hypoxia-induced 5'-UTR m^6A (**f, h**).

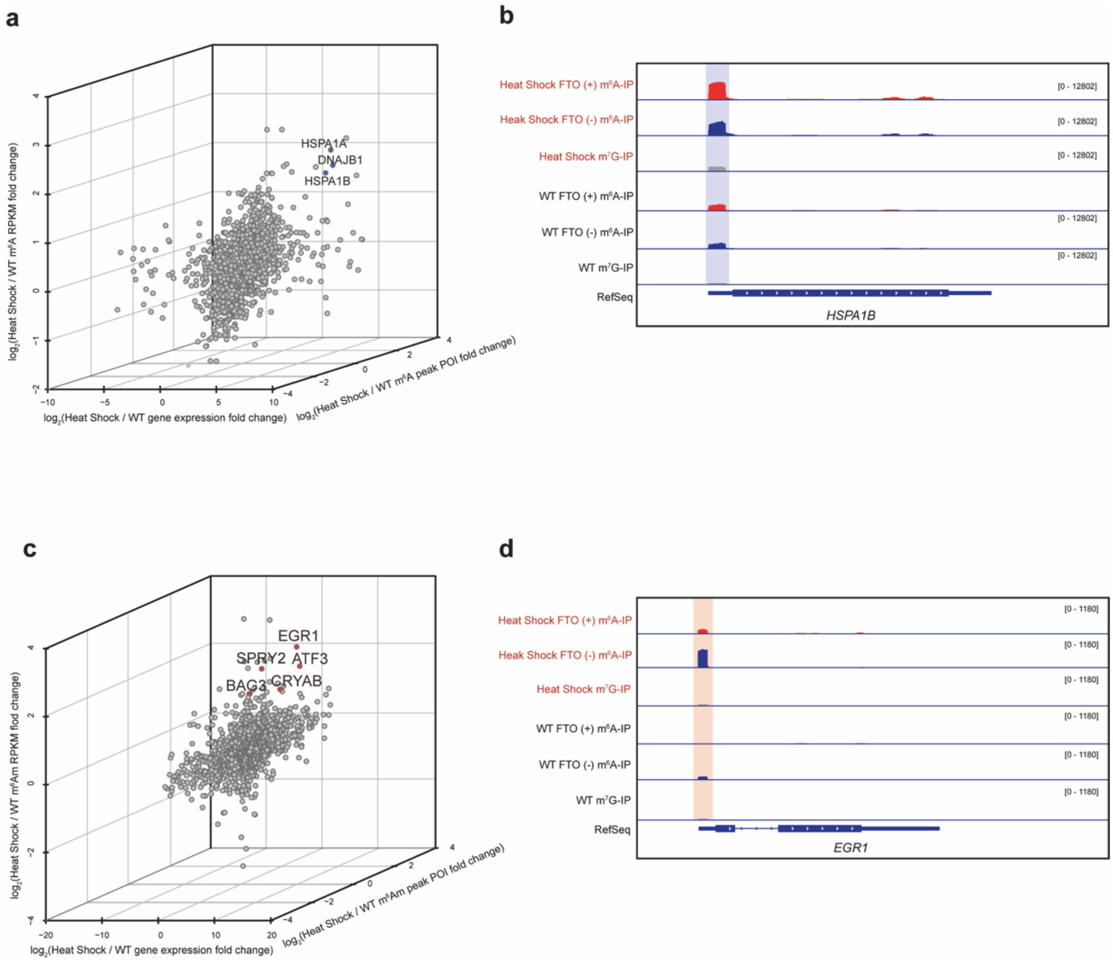

Supplementary Figure 10. Altered 5'-UTR m^{6}A and m^{6}Am methylome in response to heat shock. (**a**) A 3D plot depicting fold changes of gene expression, 5'-UTR m^{6}A intensity, and 5'UTR m^{6}A POI score after heat shock stress. Several typical genes were highlighted. (**b**) A representative view showing increased 5'-UTR m^{6}A in *HSPA1B* after heat shock. (**c**) A 3D plot depicting fold changes of gene expression, m^{6}Am intensity, and m^{6}Am POI score after heat shock stress. Several typical genes were highlighted. (**d**) A representative view showing increased m^{6}Am, but not m^{6}A, in *EGR1* after heat shock. Pink and blue background colors denote m^{6}Am and m^{6}A signals, respectively.

a

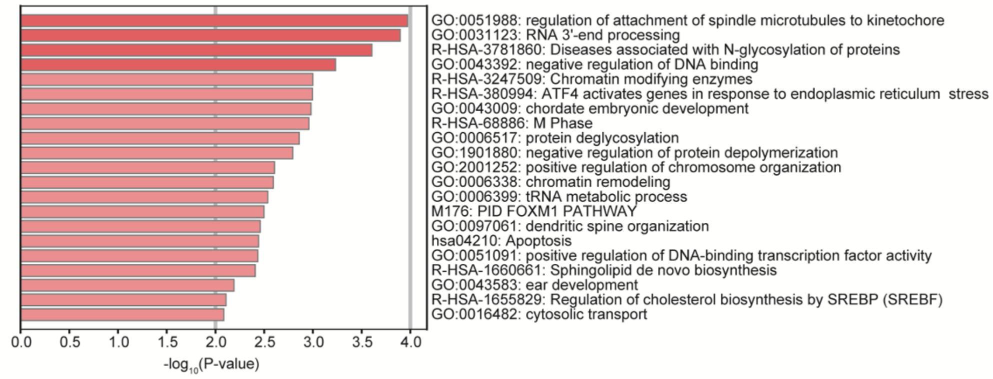

b

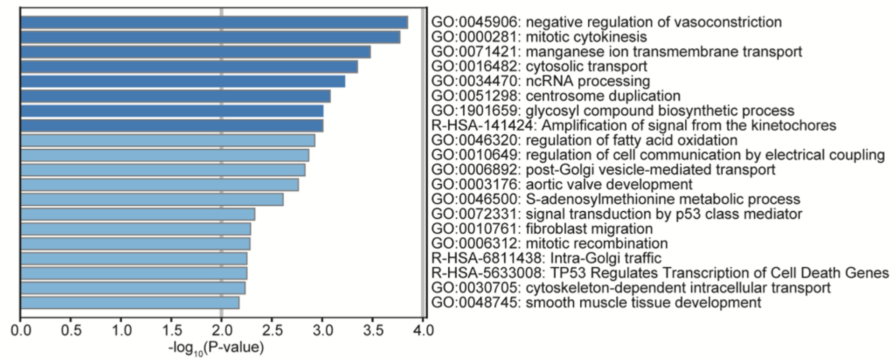

Supplementary Figure 11. GO analysis of hypoxia-inducible m^{6}Am or 5'-UTR m^{6}A marked genes. GO analysis was performed for the human transcripts containing hypoxia-inducible m^{6}Am (**a**) or 5'-UTR m^{6}A (**b**), using DAVID database (*P*-value provided by DAVID).
