# Supplementary material for: m6Am-seq reveals the dynamic m6Am methylation in the human transcriptome
Source: Nat Commun. 2021 Aug 6;12:4778. doi: 10.1038/s41467-021-25105-5 (PMC8346571; doi:10.1038/s41467-021-25105-5)
Supplement: Supplementary file 2 — Reporting Summary [file 41467_2021_25105_MOESM2_ESM.pdf]

nature portfolio

Corresponding author(s): Chengqi Yi

Last updated by author(s): Jul 18, 2021

Reporting Summary

Nature Portfolio wishes to improve the reproducibility of the work that we publish. This form provides structure for consistency and transparency in reporting. For further information on Nature Portfolio policies, see our Editorial Policies and the Editorial Policy Checklist.

Statistics

For all statistical analyses, confirm that the following items are present in the figure legend, table legend, main text, or Methods section.

n/a	Confirmed	
[ ]	[x]	The exact sample size (n) for each experimental group/condition, given as a discrete number and unit of measurement
[ ]	[x]	A statement on whether measurements were taken from distinct samples or whether the same sample was measured repeatedly
[ ]	[x]	The statistical test(s) used AND whether they are one- or two-sided<br>*Only common tests should be described solely by name; describe more complex techniques in the Methods section.*
[x]	[ ]	A description of all covariates tested
[x]	[ ]	A description of any assumptions or corrections, such as tests of normality and adjustment for multiple comparisons
[ ]	[x]	A full description of the statistical parameters including central tendency (e.g. means) or other basic estimates (e.g. regression coefficient) AND variation (e.g. standard deviation) or associated estimates of uncertainty (e.g. confidence intervals)
[ ]	[x]	For null hypothesis testing, the test statistic (e.g. F, t, r) with confidence intervals, effect sizes, degrees of freedom and P value noted<br>*Give P values as exact values whenever suitable.*
[x]	[ ]	For Bayesian analysis, information on the choice of priors and Markov chain Monte Carlo settings
[x]	[ ]	For hierarchical and complex designs, identification of the appropriate level for tests and full reporting of outcomes
[ ]	[x]	Estimates of effect sizes (e.g. Cohen's d, Pearson's r), indicating how they were calculated

Our web collection on statistics for biologists contains articles on many of the points above.

Software and code

Policy information about availability of computer code

Data collection	Next-generation sequencing data was collected and demultiplexed by Illumina HiSeq X-Ten.
Data analysis	Trim_galore (version 0.6.6)<br>Cutadapt (version 1.18)<br>HISAT2 (version 2.1.0)<br>TopHat2 (version 2.0.13)<br>Cufflinks (version 2.2.1)<br>samtools (version 1.9)<br>homer(version 4.10)<br>MACS2 (version 2.1.1)<br>MEME (version 4.12.0)<br>DESeq2 (version 3.11)<br>exomePeak (version 2.13)<br>R environment (version 3.6)<br>DAVID (version 6.8)<br>Deeptools (version 3.5.0)<br>All custom code are available at https://github.com/KaiLi-bioinfo/m6Am_seq/

For manuscripts utilizing custom algorithms or software that are central to the research but not yet described in published literature, software must be made available to editors and reviewers. We strongly encourage code deposition in a community repository (e.g. GitHub). See the Nature Portfolio guidelines for submitting code & software for further information.

Data

Policy information about availability of data

All manuscripts must include a data availability statement. This statement should provide the following information, where applicable:

- Accession codes, unique identifiers, or web links for publicly available datasets
- A description of any restrictions on data availability
- For clinical datasets or third party data, please ensure that the statement adheres to our policy

The sequence data generated in this study have been deposited in the NCBI GEO, under accession code GSE180253 that is publicly accessible at https://www.ncbi.nlm.nih.gov/geo/query/acc.cgi?acc=GSE180253. For H3K27ac, H3K4me3, H3K4me1 and H3K9me3 chip-seq public data was downloaded from ENCODE portal (https://www.encodeproject.org/) with the following identifiers: ENCSR000DTU, ENCSR000FCJ, ENCSR000FCH, ENCSR000FCG. GRO-seq data was downloaded from GEO database (GSE92375).

Field-specific reporting

Please select the one below that is the best fit for your research. If you are not sure, read the appropriate sections before making your selection.

☒ Life sciences ☐ Behavioural & social sciences ☐ Ecological, evolutionary & environmental sciences

For a reference copy of the document with all sections, see nature.com/documents/nr-reporting-summary-flat.pdf

Life sciences study design

All studies must disclose on these points even when the disclosure is negative.

Sample size	For experiments with analysis, a minimum of 2 biological replicates were performed to confirm reproducibility.
Data exclusions	No data was excluded.
Replication	All experiments were carried out in at least 2 biological replicates. All attempts to replicate data were successful.
Randomization	Cells were randomly assigned to a given treatment or stress group. RNA was randomly assigned to a demethylase treatment or inactivated-demethylase treatment.
Blinding	Blinding was not performed as experimental conditions and samples treatment were evident.

Reporting for specific materials, systems and methods

We require information from authors about some types of materials, experimental systems and methods used in many studies. Here, indicate whether each material, system or method listed is relevant to your study. If you are not sure if a list item applies to your research, read the appropriate section before selecting a response.

Materials & experimental systems

n/a	Involved in the study	
☐	☒	Antibodies
☐	☒	Eukaryotic cell lines
☒	☐	Palaeontology and archaeology
☒	☐	Animals and other organisms
☒	☐	Human research participants
☒	☐	Clinical data
☒	☐	Dual use research of concern

Methods

n/a	Involved in the study	
☒	☐	ChIP-seq
☒	☐	Flow cytometry
☒	☐	MRI-based neuroimaging

Antibodies

Antibodies used	Anti-N6-methyladenosine (m6A) Antibody (Millipore; cat#ABE572; polyclonal; rabbit)<br>Anti-7-methylguanosine (m7G)-Cap mAb (MBL; cat#RN016M; monoclonal; Clone 150-15; mouse)
Validation	For m6A antibody, we have previously validated this antibody for IP in human cells (Liu et al, Molecular Cell, ref.21).<br>The m7G antibody has been validated by the manufacturer (https://www.mblbio.com/bio/g/dtl/A/index.html?pcd=RN016M) and a previous study (https://academic.oup.com/nar/article/49/10/5520/6274539).

Eukaryotic cell lines

Policy information about cell lines

Cell line source(s)	HEK293T (ATCC)
Authentication	HEK293T cells were authenticated by RNA-seq. PCIF1-depleted cells were validated by western blot and RNA-seq.
Mycoplasma contamination	The cells used here tested negatively for mycoplasma contamination. Mycoplasma contamination was tested by PCR.
Commonly misidentified lines (See ICLAC register)	No commonly misidentified cell lines were used.
