# Supplementary material for: m6Am-seq reveals the dynamic m6Am methylation in the human transcriptome
Source: Nat Commun. 2021 Aug 6;12:4778. doi: 10.1038/s41467-021-25105-5 (PMC8346571; doi:10.1038/s41467-021-25105-5)
Supplement: Supplementary file 9 — Description of Additional Supplementary Files [file 41467_2021_25105_MOESM9_ESM.pdf]

Title: Supplementary Data 1.

Description: m^6Am-seq identified 1,652 high confidence m^6Am peaks from 1,635 genes in the human transcriptome.

Title: Supplementary Data 2.

Description: m^6Am-seq identified 1,307 5'-UTR m^6A peaks from 1,297 genes in the human transcriptome.

Title: Supplementary Data 3.

Description: m^6Am-seq identified 2,166 m^6Am sites from 1,459 genes.

Title: Supplementary Data 4.

Description: Heat shock stress-inducible m^6Am and 5'-UTR m^6A peaks.

Title: Supplementary Data 5.

Description: Hypoxia stress-inducible m^6Am and 5'-UTR m^6A peaks.

Title: Supplementary Data 6.

Description: Primer sequences for designed spike-in model sequences.
